# Genome sequencing and comparative analysis of three *Chlamydia pecorum* strains associated with different pathogenic outcomes

**DOI:** 10.1186/1471-2164-15-23

**Published:** 2014-01-14

**Authors:** Michelle Sait, Morag Livingstone, Ewan M Clark, Nick Wheelhouse, Lucy Spalding, Bryan Markey, Simone Magnino, Frederick A Lainson, Garry SA Myers, David Longbottom

**Affiliations:** Moredun Research Institute, Pentlands Science Park, Bush Loan, Edinburgh, Midlothian, EH26 0PZ UK; School of Veterinary Medicine, College of Agriculture, Food Science and Veterinary Medicine, University College Dublin, Belfield, Dublin 4, Ireland; Istituto Zooprofilattico Sperimentale della Lombardia e dell’Emilia Romagna “Bruno Ubertini”, National Reference Laboratory for Animal Chlamydioses, Sezione Diagnostica di Pavia, Strada Campeggi 61, 27100 Pavia, Italy; Institute for Genome Sciences, University of Maryland School of Medicine, Baltimore, MD USA; Microbiological Diagnostic Unit, The University of Melbourne, Parkville, Victoria 3010 Australia; BigDNA Ltd, Wallace Building, Roslin BioCentre, Roslin, Midlothian, EH25 9PP UK

**Keywords:** *Chlamydia pecorum*, Genome sequence, Polymorphic membrane proteins, Plasticity zone, Tryptophan metabolism, Folate biosynthesis, Clustered tandem repeats

## Abstract

**Background:**

*Chlamydia pecorum* is the causative agent of a number of acute diseases, but most often causes persistent, subclinical infection in ruminants, swine and birds. In this study, the genome sequences of three *C. pecorum* strains isolated from the faeces of a sheep with inapparent enteric infection (strain W73), from the synovial fluid of a sheep with polyarthritis (strain P787) and from a cervical swab taken from a cow with metritis (strain PV3056/3) were determined using Illumina/Solexa and Roche 454 genome sequencing.

**Results:**

Gene order and synteny was almost identical between *C. pecorum* strains and *C. psittaci*. Differences between *C. pecorum* and other chlamydiae occurred at a number of loci, including the plasticity zone, which contained a MAC/perforin domain protein, two copies of a >3400 amino acid putative cytotoxin gene and four (PV3056/3) or five (P787 and W73) genes encoding phospholipase D. *Chlamydia pecorum* contains an almost intact tryptophan biosynthesis operon encoding *trpABCDFR* and has the ability to sequester kynurenine from its host, however it lacks the genes *folA*, *folKP* and *folB* required for folate metabolism found in other chlamydiae. A total of 15 polymorphic membrane proteins were identified, belonging to six pmp families. Strains possess an intact type III secretion system composed of 18 structural genes and accessory proteins, however a number of putative inc effector proteins widely distributed in chlamydiae are absent from *C. pecorum*. Two genes encoding the hypothetical protein ORF663 and IncA contain variable numbers of repeat sequences that could be associated with persistence of infection.

**Conclusions:**

Genome sequencing of three *C. pecorum* strains, originating from animals with different disease manifestations, has identified differences in ORF663 and pseudogene content between strains and has identified genes and metabolic traits that may influence intracellular survival, pathogenicity and evasion of the host immune system.

**Electronic supplementary material:**

The online version of this article (doi:10.1186/1471-2164-15-23) contains supplementary material, which is available to authorized users.

## Background

Members of the genus *Chlamydia* are Gram-negative, obligate intracellular pathogens that share a biphasic developmental cycle. *Chlamydia pecorum* infects a broad host range, including small and large ruminants, swine, birds and marsupials. Seroprevalence and PCR-based studies suggest that infection or exposure to *C. pecorum* and/or *C. abortus* is almost ubiquitous in cattle and sheep [1–5]. In the majority of these cases, infection is subclinical, with *C. pecorum* being routinely detected in the intestine and genital tract. The incidence and severity of disease caused by *C. pecorum* appears to be heightened in koalas and is associated with clinical disease such as conjunctivitis, urinary- and reproductive tract disease, and infertility [6]. Many chlamydial species, including *C. pecorum* can enter persistent states, characterised *in vitro* by enlarged, morphologically aberrant, non-fusogenic reticulate bodies (RBs). Persistence can be induced *in vitro* by antibiotic exposure [7], amino acid- [8] or iron- [9] deficiencies and exposure to IFN-γ [10] and it is likely that *C. pecorum* causes a persistent, subclinical infection in the host. Subclinical infections can have detrimental effects on the animal’s health. Animals with inapparent chlamydiae infections have higher body temperatures, lower body weights, reduced growth rates, reduced iron, haemoglobin, haematocrit and leukocyte levels and a higher incidence of follicular bronchiolitis [11–13]. *C. pecorum* can also cause clinical disease including encephalomyelitis, vaginitis, endometritis, mastitis, conjunctivitis, polyarthritis, pneumonia, enteritis, orchitis, pleuritis, infertility or pericarditis [6].

Genetic variation has been reported to occur between *C. pecorum* strains in *ompA*, the *rrn*-*nqr*F intergenic region, *incA*, rRNAs, a number of housekeeping genes and the hypothetical protein ORF663 [14–22]. These and other unidentified genomic differences may enable differentiation between strains isolated from asymptomatic or diseased animals. However, to date, only the genome sequence of a single *C. pecorum* strain (E58) has been published [23]. The genetic factors responsible for the diverse host range, tissue tropism, disease outcomes and associated sequelae of *C. pecorum* infections are thus still poorly understood. In this study, we present the complete genome sequences of three *C. pecorum* strains isolated from animals exhibiting different disease manifestations and use comparative genomics to provide insights into the biology of *C. pecorum* and to identify both genus- and species-specific virulence factors.

## Results and discussion

### Genome features and comparative analysis

**Figure 1:**
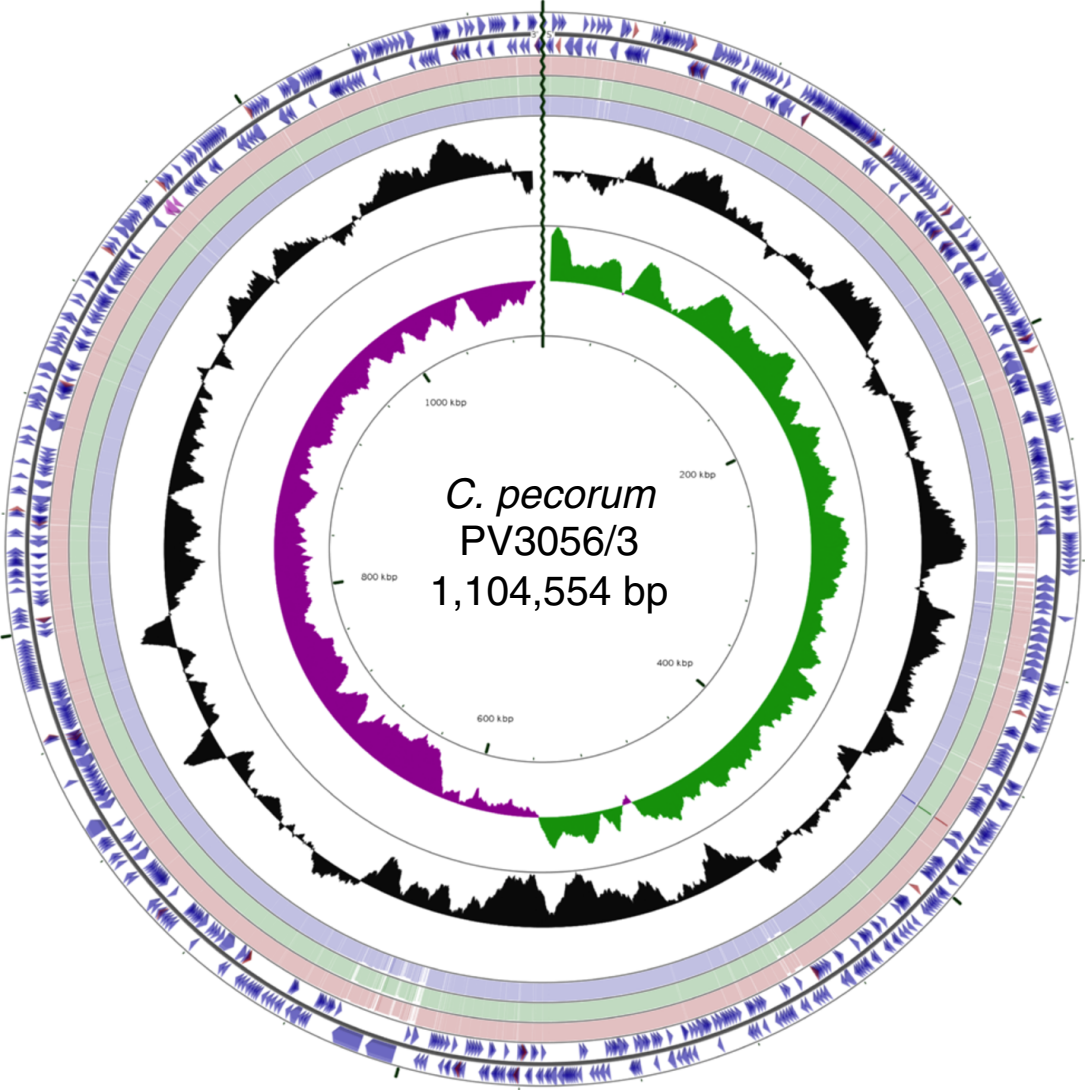
**Circular representation of the genome of** ***C. pecorum*** **PV3056/3.** Circles from the outside in show: the positions of protein-coding genes (blue), tRNA genes (red) and rRNA genes (pink) on the positive (circle 1), and negative (circle 2), strands respectively. Circles 3–5 show the positions of BLAST hits detected through blastn comparisons of PV3056/3 against W73 (circle 3), P787 (circle 4) and E58 (circle 5) with the following settings: query split size = 50,000 bp, query split overlap size =0, expect value cutoff =0.00001. Low complexity sequences were eliminated from the analysis. The height of the shading in the BLAST results rings is proportional to the percent identity of the hit. Overlapping hits appear as darker shading. Circles 6 and 7 show plots of GC content and GC skew plotted as the deviation from the average for the entire sequence. The origin of replication is indicated by the vertical zig-zag line.

The genomes of *C. pecorum* PV3056/3 (CPE1), W73 (CPE2) and P787 (CPE3) each comprise a single circular chromosome of 1,104,552 bp, 1,106,534 bp and 1,106,412 bp, respectively. The general features of these genomes compared to reference strain E58 [GenBank: CP002608] [23] are shown in Figure 1 and Table 1. The G + C content of each genome is 41.1% and none of the strains contain any plasmids. The origins of replication were assigned based on base composition asymmetry of the genomes and in each genome the oriC is located upstream of the *hem*B gene. There are 38 tRNA genes corresponding to all the amino acids except selenocysteine and pyrrolysine, one rRNA operon, and 3 sRNA molecules corresponding to SsrA, RNaseP and ffs (Additional file 1: Table S1) present in each chromosome. Annotation identified 927 (PV3056/3) and 928 (P787 and W73) predicted coding sequences (CDSs), representing a coding density of 92.5%. Of the predicted CDSs, 628 (67.7%, PV3056/3), 630 (67.9%, W73) and 629 (67.8%, P787) were functionally assigned based on previous experimental evidence or database similarity and motif matches. For hypothetical proteins with no functional assignment, 209 (PV3056/3) and 208 (P787 and W73) proteins (69.8%) were either unique to *C. pecorum* or significantly similar to proteins from chlamydial species. The number of pseudogenes varied between *C. pecorum* strains, with the majority occurring due to frameshift mutations in homopolymeric tracts. PV3056/3 contained 6 pseudogenes, while P787 and W73 contained 3 pseudogenes each. Pseudogenes were annotated as phospholipase D family proteins, an ABC transporter protein and hypothetical proteins (Additional file 1: Table S2).

**Table 1:** **General features of** ***C. pecorum*** **PV3056/3, P787 and W73 compared with the type strain E58 [CP002608**]

	***C. pecorum*** (PV3056/3)	***C. pecorum*** (P787)	***C. pecorum*** (W73)	***C. pecorum*** (E58) [ [23] ]
Date of isolation	1991	1977	1989	Circa 1940
Country	Italy	Scotland	Northern Ireland	USA
Source	Cow, cervical swab	Sheep, synovial fluid	Sheep, faeces	Calf, brain
Disease pathotype	Metritis	Polyarthritis	Asymptomatic/enteric	Encephylomyelitis
Genome size (bp)	1,104,552	1,106,412	1,106,534	1,106,197
% GC of genome	41.1	41.1	41.1	41.1
% coding	91.9	92.1	92.1	91.9
Predicted CDS	927	928	928	1073
Predicted no. of pseudogenes	6	3	3	1
No. of CDS with functional assignment	628 (67.7%)	629 (67.8%)	630 (67.9%)	-
No. of pmp proteins	15	15	15	15
No. of tRNA genes	38	38	38	38
No. of rRNA operons	1	1	1	1
No. of sRNA molecules	3	3	3	3
Location of OriC region	1104229–147 nt (471 nt)	1106263–147 nt (296 nt)	1106389–147 nt (295 nt)	305941–306155 nt (215 nt)

**Figure 2:**
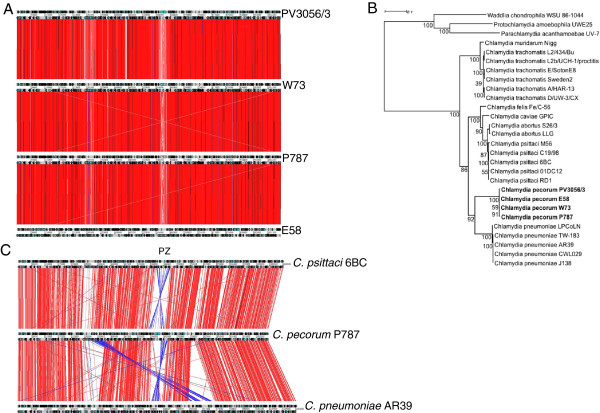
**Comparative analysis of** ***C. pecorum*** **. (A)** Whole genome comparison between *C. pecorum* strains PV3056/3, P787 and W73 and reference strain E58 [CP002608] showing ACT comparison of amino acid matches between complete 6-frame translations (computed using tblastx). Grey bars represent the forward and reverse strands of DNA with CDSs marked as arrows. The scale is marked in base pairs. The red bars represent individual tblastx matches. Inverted matches are coloured blue. **(B)** Maximum-likelihood (PhyML) phylogenetic tree calculated using a JTT + G substitution model with the concatenated sequences of 48 ribosomal proteins from species of the family Chlamydiaceae (an outgroup comprising sequences from other Chlamydiales family members is included). Bootstrap proportion values are indicated at the node. The scale bar indicates 0.1 expected substitutions per site. **(C)** Whole genome comparison of amino acid matches between *C. pecorum* PV3056/3, *C. psittaci* 6BC [CP002586] and *C. pneumoniae* AR39 [AE002161] showing ACT comparison of amino acid matches between complete 6-frame translations (computed using tblastx). Grey bars represent the forward and reverse strands of DNA with CDS encoding products marked as black arrows. The scale is marked in base pairs. The red bars represent individual tblastx matches. Inverted matches are coloured blue. The plasticity zone (PZ) is indicated.

**Figure 3:**
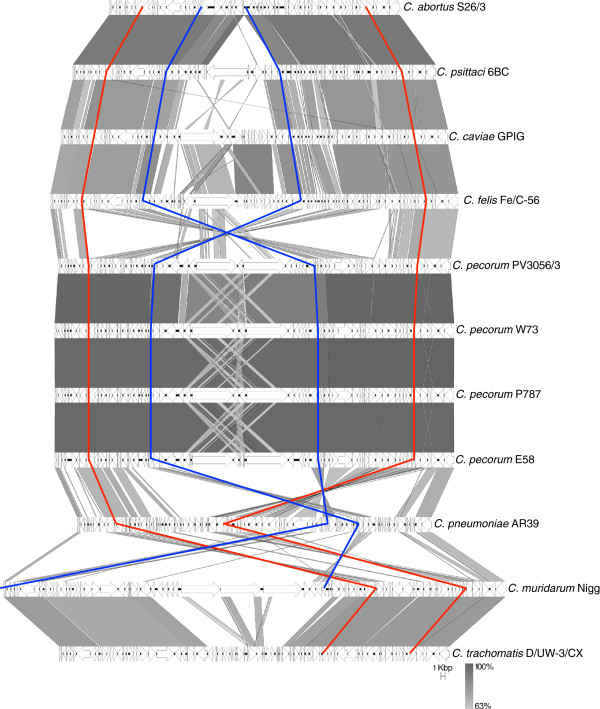
**Comparison of genomic regions flanking the** ***C. pecorum*** **plasticity zone.** Comparison of nucleotide matches (computed using blastn) between the genes encoding glucose-6-phosphate 1-dehydrogenase and a peptide ABC transporter (indicated by red vertical lines) for available representative genomes of the family Chlamydiaceae. CDSs are marked as arrows. The blue vertical line represents the plasticity zone defined as the regions between *acc*B and *gua*B. The depth of shading is indicative of the percentage blastn match, as indicated bottom right. The scale is marked in kilobase pairs.

Comparative analysis of the three *C. pecorum* genomes to reference strain E58 [GenBank: CP002608] [23] revealed a high level of sequence conservation, gene content and order (Figure 2A). Phylogenetic analysis of 48 concatenated ribosomal proteins from *Chlamydia* species revealed *C. pecorum* strains to be most closely related to *C. pneumoniae* (Figure 2B), an observation in agreement with the MLST analysis of several housekeeping genes [24]. However, global comparisons between *C. pecorum* and other chlamydial species reveal gene order and synteny to be most similar to *C. psittaci* (Figure 2C). Comparisons between *C. pecorum* P787, *C. psittaci* 6BC [GenBank: CP002586] and *C. pneumoniae* AR39 [GenBank: AE002161] show chromosomal rearrangements including a large DNA inversion in the plasticity zone (PZ) of the genome. An additional asymmetrical translocation is observed between *C. pecorum* and *C. pneumoniae* in the region flanking the *pmpG* genes corresponding to the region 322207–381219 in P787 and encoding 55 genes (CPE3_0288-CPE3_0342) (Figure 2C). Comparative analysis between *C. pecorum* and other chlamydial species suggests that genetic rearrangements also occur in the regions flanking the PZ between the conserved orthologs *zwf,* encoding glucose-6-phosphate 1-dehydrogenase (CPE1_0526, CPE2_0526, CPE3_0526) and a peptide ABC transporter (CPE1_0575, CPE2_0576, CPE3_0576) spanning a 72.0-73.7 kb region encoding 46 (PV3056/3) and 47 (W73 and P787) genes (Figure 3).

### Metabolic characteristics

Comparative genomics of chlamydial species has identified a number of genes coding for metabolic functions, such as tryptophan metabolism, biotin biosynthesis and folate biosynthesis, where subtle variations in gene content may contribute to growth of the organism *in vivo* and the ability to evade the host immune system [23, 25–29].

**Figure 4:**
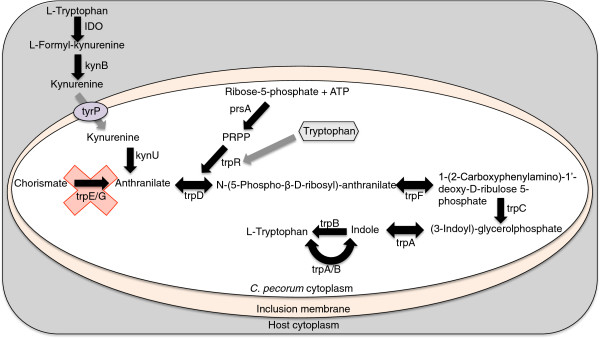
**Schematic diagram showing the genes in** ***C. pecorum*** **and the host-cell involved in tryptophan metabolism.**

The genome of *C. pecorum* contains an almost intact tryptophan biosynthesis operon, consisting of anthranilate phosphoribosyltransferase (*trpD*), phosphoribosylanthranilate isomerase (*trpF*), indole-3-glycerol phosphate synthase (*trpC*), tryptophan synthase alpha chain (*trpA*) and tryptophan synthase beta chain (*trpB*) genes. This complement of genes and the gene arrangement is most similar to that found in *C. caviae*, however the tryptophan biosynthesis operon in *C. pecorum* is not located in the plasticity zone and does not contain the additional *trpB* gene found in *C. caviae*[26]. The complement of genes observed in *C. pecorum* would theoretically permit the production of tryptophan from the substrate anthranilate. However, the gene complement will not permit the first step of tryptophan biosynthesis, the conversion of chorismate to anthranilate, which is catalysed by anthranilate synthetase (trpE/G). The acquisition of anthranilate could be achieved by *C. pecorum* through the direct uptake of kynurenine from the host cell via an aromatic amino acid transporter similar to tyrP (CPE1_0759, CPE2_0760, CPE3_0760), converted to anthranilate by kynureninase (*kynU,* CPE1_0671, CPE2_0672, CPE3_0672) and further metabolised to phosphoribosyl anthranilate by trpD in the presence of PRPP synthase and then to tryptophan via a series of intermediates (Figure 4). In mammalian cells, the production of the pro-inflammatory cytokine IFN-γ by the host has been documented to decrease the availability of L-tryptophan in host cells by the induction of indoleamine 2,3-dioxygenase (IDO) that converts L-tryptophan to L-formylkynurenine and then subsequently to kynurenine by arylformamidase [30]. This limitation of tryptophan by the host can lead either to the resolution of chlamydial infections or the establishment of persistent infections by chlamydial species [31]. The ability of *C. pecorum* to synthesise tryptophan in an IFN-γ rich environment may contribute to its ability to form persistent, subclinical infections.

The 3 sequenced *C. pecorum* strains and E58 contain the biotin biosynthesis operon encoding *bioBFDA*. (CPE1_0687-CPE1_0690; CPE2_0688-CPE2_0691; CPE3_0688-CPE3_0691). This region shows significant variability between chlamydial species, being absent in *C. caviae*, *C. trachomatis* and *C. muridarum* but present in *C. abortus*, *C. psittaci*, *C. felis* and *C. pneumoniae*. The ability to synthesise biotin is hypothesised to assist in the colonization of biotin-limited niches and contribute to the tissue tropism differences observed in the chlamydiae [25]. Upstream of *bioBFDA,* located between *dapB* and *bioB*, a series of genes encoding hypothetical proteins with unknown function and limited distribution across chlamydial species are present. *Chlamydia abortus*, *C. psittaci* and *C. felis* genomes contain four genes (in *C. abortus*, CAB681, CAB682, CAB683 and CAB684), *C. pneumoniae* contains 2 genes in this region that are homologues of CAB681 and CAB682, while *C. pecorum* contains one gene in this region that is homologous to CAB684 (Additional file 2: Figure S1).

**Figure 5:**
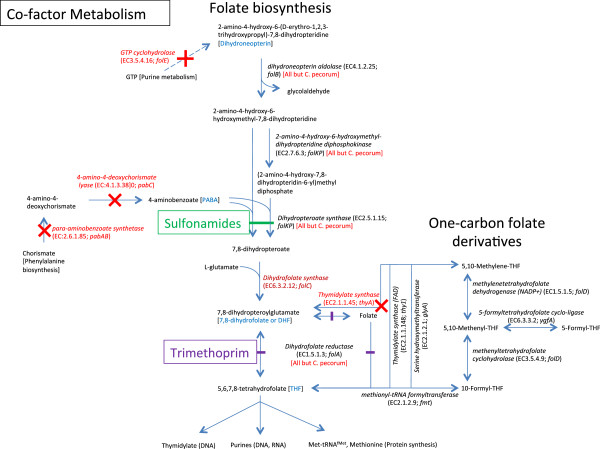
**Schematic diagram showing the genes involved in folate biosynthesis and one-carbon pool folate derivative metabolism present in** ***C. pecorum*** **and other chlamydial species.**

Three genes encoding key enzymes involved in folate biosynthesis, namely dihydrofolate reductase (*folA*), dihydropteroate synthase (*folKP*) and dihydroneopterin aldolase (*folB*), are absent from all 4 *C. pecorum* genomes (Figure 5). These genes are present in other chlamydiaceae species (*C. abortus*, *C. psittaci*, *C. caviae*, *C. felis*, *C. pneumoniae*, *C. muridarum* and *C. trachomatis*) (Figure 5, Additional file 1: Table S3). These findings suggest that *C. pecorum* will be unable to synthesize folate or 7,8-dihydrofolate (DHF) and may require an exogenous source. In members of the *Firmicutes*, this is achieved through active transport systems [32], however homologues to these could not be identified in *C. pecorum*. The absence of genes *folA* and *folKP* in *C. pecorum* would theoretically confer a natural resistance to trimethoprim and sulphonamide antibiotics, which act as substrate analogues of dihydrofolate reductase (FolA) and dihydropteroate synthase (FolKP), respectively. The absence of genes *thyA* (classical thymidylate synthase) and *folA* in all *C. pecorum* genomes indicates that the formation of 5,6,7,8-tetrahydrofolate (THF), an essential donor of one-carbon units for DNA, RNA and protein syntheses, must be achieved through other pathways. Indeed, all Chlamydiaceae species sequenced to date, including *C. pecorum*, contain homologs for *thyX* (also known as *thy1*), *glyA*, *folD*, *ygfA* and *fmt* that encode enzymes allowing the synthesis and interconversion of carbon-one folate derivatives (Figure 5, Additional file 1: Table S3) in the production of dTMP (thymidylate; required for DNA synthesis) and formylmethionine (initiator methionine for protein synthesis). The flavin-dependent alternative thymidylate synthase ThyX uses 5,10-methylenetetrahydrofolate as a one-carbon donor and links dTMP catalysis with the formation of THF [33]. However, bacteria with a *thyX*^+^/*folA*^-^/*thyA*^*-*^ genotype, like *C. pecorum*, must still contain reduced folates for RNA and protein synthesis to take place. This is likely achieved through an alternate pathway involving other enzymes encoded by *glyA* (serine hydroxymethyltransferase), *folD* (methylene tetrahydrofolate cyclohydrolase/dehydrogenase), *ygfA* (5-formyltetrahydrofolate cyclo-ligase) and *fmt* (methionyl-tRNA formyltransferase). The novel folate cycle observed in *C. pecorum* may contribute to the occurrence of persistent infections due to the limited pool of reduced folates available to the cell. As *C. pecorum* is likely to acquire folate directly from the host cell, increased competition could result in folate deficiency in the host, contributing to the increased levels of anaemia and lower body weights observed in infected animals [11, 13].

### Bacterial secretion systems

**Figure 6:**
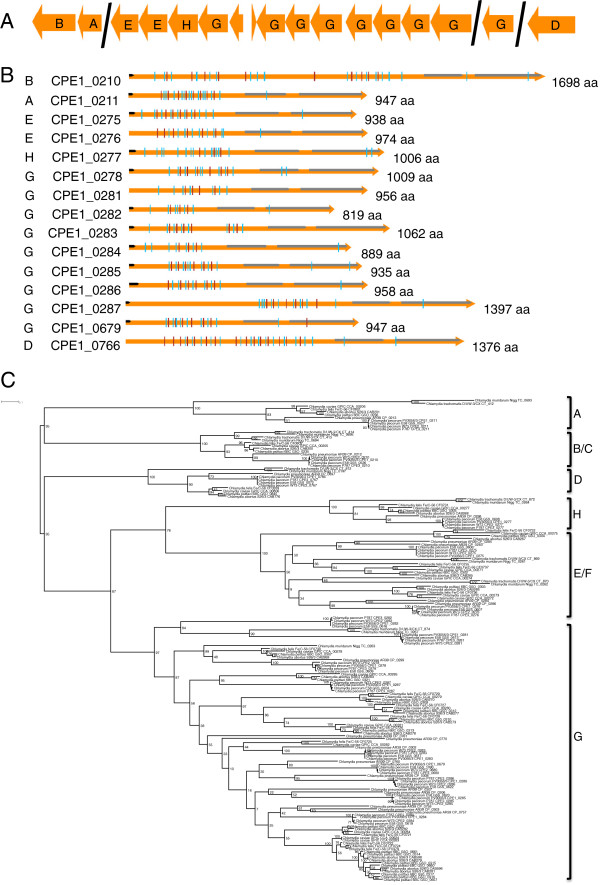
**Polymorphic membrane proteins in** ***C. pecorum*** **. (A)** Genetic organisation of Pmps in *C. pecorum* with gene families (as indicated) identified following BLAST and phylogenetic comparison (see **C**. below) with other published Pmps. **(B)** Schematic diagram showing the conserved Pmp features, comprising: predicted pmpM and autotransporter domains (grey arrows); predicted pmp passenger domain repeat motifs GG[A/L/V/I][I/L/V/Y] (blue vertical lines); and FXXN motifs (red vertical lines). Signal peptide sequences are as indicated (black arrows). The predicted number of amino acids (aa) is indicated to the right of the gene. Gene families (see **C**. below) are indicated to the left of the locus tags. **(C)** Maximum-likelihood (PhyML) phylogenetic tree of autotransporter domains showing clustering of Pmps into gene families (indicated to the right of the groups). For clarity the figure shown displays a subset of 121 sequences based on a larger alignment of 367 sequences. Trees were calculated using a JTT + I + G substitution model. Bootstrap support is indicated by number at the node. The scale bar indicates 0.1 expected substitutions per site.

The *C. pecorum* genomes each contain 15 genes that encode members of the type V “autotransporter (AT)” secretion system (Figure 6A). In chlamydial species, these are referred to as polymorphic membrane proteins (pmps) and are present in all sequenced genomes, in numbers ranging from 9 in *C. trachomatis* to 21 in *C. pneumoniae. C. pecorum* ATs range in predicted size and pI from 89 to 176 kDa and 5.05 to 8.93 respectively (Additional file 1: Table S4) and possess the conserved AT domain architecture comprising a central pmpM domain, C-terminal autotransporter (AT) domain and predicted passenger-domains with a variable number of the repeat motifs GG[A/L/V/I][I/L/V/Y] and FXXN [34]. N-terminal signal sequences with potential signal peptidase 1 cleavage sites were identified in 12 ATs (Figure 6B, Additional file 1: Table S4). Phylogenetic analysis of the C-terminal AT domains identified the *C. pecorum* ATs as belonging to 6 gene families. Individual gene families showed high bootstrap support (>97%) but only weak support at deeper branches (35-87%) (Figure 6C). Phylogenetic network analysis performed on AT sequences show separation into the AT gene families but suggests that recombination is occurring between AT domains (phi test for recombination p = 0.02173) (Additional file 2: Figure S2). ATs were located in 4 genetic loci consisting of two singletons belonging to the pmpD (CPE1_0766, CPE2_0767, CPE3_0767) and pmpG (CPE1_0679, CPE2_0680, CPE3_0680) protein families, two pairs of genes belonging to pmpB and pmpA (CPE1_0210, CPE2_0210, CPE3_0210; CPE1_0211, CPE2_0211, CPE3_0211), and a cluster of 11 genes belonging to the pmpE (CPE1_0275-0276, CPE2_0275-0276, CPE3_0275-0276), pmpH (CPE1_0277, CPE2_0277, CPE3_0277) and pmpG (CPE1_0278, CPE1_0281-0287, CPE2_0278, CPE2_0281-0287, CPE3_0278, CPE3_0281-0287) protein families (Figure 6A, 6C, Additional file 2: Figure S2). All AT-encoding genes were intact in *C. pecorum* except for the gene encoding pmpA in E58 (G5S_0527). Based on the short length of homopolymeric tracts identified in *C. pecorum* ATs (maximum 8 nucleotides), it appears less likely that expression of these genes are subject to phase variation by strand slippage mechanisms compared to ATs from other organisms such as *C. abortus* (maximum 16 nucleotides).

The Type III secretion system (T3SS) consists of 18 genes encoding the major structural components of the secretion apparatus, accessory proteins and chaperones and is arranged in 4 genetic loci (Additional file 1: Table S5). In sequenced chlamydial genomes, a number of putative T3SS effector proteins belonging to the Inc or transmembrane head (TMH) protein family are located in the region extending between pmpD and lpxB (Additional file 2: Figure S3). The distance between the 3′ ends of these genes in *C. pecorum* is ~2.8 kb (2 genes) compared to 18.1 kb *C. abortus* (11 genes)*,* 17.7 kb in *C. psittaci* (11 genes)*,* 16.4 kb in *C. caviae* (13 genes), 15.9 kb in *C. felis* (11 genes) and 1.7 kb in *C. pneumoniae* (1 gene). The two genes present in this region in *C. pecorum* (CPE1_0764 (pseudogene), CPE1_0765, CPE2_0765, CPE2_0766, CPE3_0765, CPE3_0766) possess an N-terminal signal sequence, a single N-terminal transmembrane domain and two domains of unknown function (DUF1539 and DUF1548). Members of this protein family are present in *C. abortus*, *C. psittaci*, *C. caviae* and *C. felis* (3 CDSs each) and *C. pneumoniae* (1 CDS).

### Simple sequence repeats

A region of variability between *C. pecorum* and other chlamydial species is located immediately upstream of the 5S rRNA gene. This region, between the 3′ ends of the 5S rRNA and *nqrF* genes range in size from 261–269 bp in *C. pecorum* to 4464 bp in *C. caviae*. In *C. caviae* this region encodes a 1291aa residue pseudogene identified as a member of the virulence-associated invasion/intimin family of outer membrane proteins of Gram-negative bacteria. The genome of *C. abortus* contains two CDSs in place of the intimin family gene in this region, encoding a conserved membrane protein and a unique hypothetical protein. In *C. psittaci, C. felis* and *C. muridarum* these two proteins are fused to encode a single hypothetical protein. In *C. pecorum* there are no predicted CDSs in this region and the intergenic region between the 5S rRNA and *nqrF* genes comprises an 8 bp simple sequence repeat sequence AAAGCACT repeated 12 (W73, PV3056/3 and E58) or 13 times (P787) (Additional file 2: Figure S4).

Clustered tandem repeat sequences (CTRs) appearing in the hypothetical protein ORF663 (CPE1_0343, CPE2_0343, CPE3_0343) have been used to differentiate between pathogenic and non-pathogenic strains of *C. pecorum* with non-pathogenic strains containing a greater number of CTRs [21]. The *C. pecorum* strains contained different numbers and types of CTRs varying from 14–27 CTRs in the *C. pecorum* strains originating from diseased animals (PV3056/3 and P787) to 52 CTRs in W73 that was isolated from an animal with subclinical disease (Table 2). Whilst no predicted function has been assigned to ORF663, N-terminal signal peptides and two transmembrane domains were identified in the corresponding genes of PV3056/3, W73 and P787 suggesting that the protein may be surface expressed. Indeed, the high proportions of serine (13.3-18.0%), proline (10.7-14.9%) and lysine (10.7-14.6%) in ORF663 could indicate adhesion functions, such as those observed in *Staphylococcus* sp. and *Streptococcus* sp. [35, 36]. In *Streptococcus* sp. correlations between the number of CTRs and pathogenicity has been reported, with deletions in the CTR causing either a loss of conformational epitopes or a decrease in the antigen size and reduction in antibody binding to the bacterial surface, resulting in increased pathogenicity [37] and it is feasible that this also occurs in chlamydiae.

**Table 2:** **Clustered tandem repeat (CTR) sequences observed in orthologs of hypothetical protein ORF663**

	CTR motif sequence															
Strain	KEPST	KEPSK	KELSP	KEPLP	KESSP	KKPSP	KEPSS	KEPSK	KSLHL	KNLHL	KNFHL	KNSHL	KNLYL	KNLQS	KEPSP	Total
PV3056/3	26	1														27
W73			27	2	15	6	1	1								52
P787			1	2			1		2	3	1	2	1	1		14
E58			7				6	1							8	22

The IncA protein is an effector protein secreted by the type III secretion system (T3SS) that is known to localize to the chlamydial inclusion membrane [38]. In *C. pecorum*, IncA has been identified as an antigen that can be used for serodiagnosis [39], and the identification and survey of CTR sequences in *incA* from isolates originating from symptomatic and asymptomatic animals suggest that the *incA* CTR motif composition in *C. pecorum* could be associated with virulence [21]. The number and composition of *incA* CTRs in the sequenced genomes varied from 8 in P787 to 12 in W73 (Table 3). This differs from those previously reported for E58 (12 × APA) and W73 (2 × APA and 8 × APAPE) [21]. The differences observed in these CTRs between strains held in different laboratories could result from adaptation of the strains to laboratory growth conditions. As IncA has been shown to contribute in establishing interactions between the inclusion and the host cell, participating in vesicle fusion or septation of the inclusion membrane during bacterial cell division [40], the presence of CTRs could contribute to the ability of *C. pecorum* to evade the host immune system or contribute to the formation of sub-clinical infections by forming non-fusogenic inclusions [21, 41].

**Table 3:** **Clustered tandem repeat sequences (CTR) observed in IncA**

		CTR motif sequence			
Strain	APA	APAE	APE	AP	Total
PV3056/3	9		1		10
W73	1	11			12
P787	2	5		1	8
E58	11	1			12

### Plasticity zone

In chlamydial species, the plasticity zone is defined as the region between inosine-5′-monophosphate dehydrogenase (*guaB*) and acetyl-CoA carboxylase (*accB*) and is the region of the genome that is most variable in gene content and sequence. In *C. pecorum*, this region is 40.3-42.1 kb in size and contains 16 (PV3056/3) or 17 (W73 and P787) genes encoding GMP synthase, an adenosine deaminase superfamily-protein, a MAC/perforin domain-containing protein, 3 (PV3056/3) or 4 (W73 and P787) phospholipase D family proteins, 2 cytotoxins and 4 hypothetical proteins (Additional file 1: Table S6).

**Figure 7:**
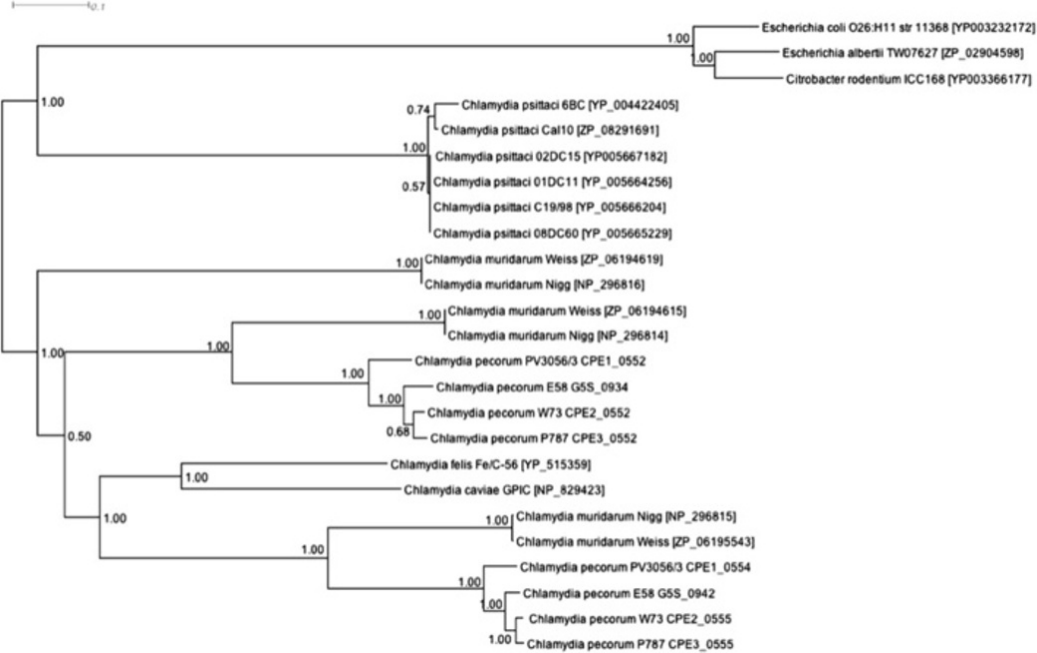
**Phylogenetic analysis of chlamydial cytotoxins.** Bayesian (MrBayes) phylogenetic tree calculated using a WAG + I + G substitution model of cytotoxin protein sequences of Chlamydiaceae species. Trees were generated using Markov Chain Monte Carlo settings of 2 runs of 625,000 generations with a burn-in of 125,000 generations with trees sampled every 100 runs. Posterior probabilities are indicated by the number at the node.

The presence of two cytotoxin genes in the PZ of each of the sequenced *C. pecorum* strains (CPE1_0552, CPE1_0554, CPE2_0552, CPE2_0555, CPE3_0552, CPE3_0555) may contribute to the ability of the organism to switch from persistent infection to causing acute disease. The cytotoxin genes share sequence similarity with *E. coli* and *Citrobacter rodentium* lymphocyte inhibitory factor A (*lifA*) and *Clostridium difficile* toxin B as well as other chlamydial cytotoxins. The 10–10.3 kb cytotoxins in *C. pecorum* consist of an N-terminal glucosyltransferase domain responsible for the biological effects of the toxin, a cysteine protease domain responsible for autocatalytic cleavage and a large domain of unknown function that may play a role in cytotoxin translocation or receptor binding. Phylogenetic analysis of cytotoxins from *C. psittaci*, *C. felis* and *C. cavie* (1 copy each), *C. pecorum* (2 copies each) and *C. muridarum* (3 copies) reveals extensive diversity within these genes (Figure 7). *C. pecorum* cytotoxins belonged to two separate gene clusters (Cluster 1:CPE1_0552, CPE2_0552, CPE3_0552; Cluster 2:CPE1_0554, CPE2_0555, CPE3_0555) each showing greatest similarity to cytotoxins from *C. muridarum*. It is unclear whether the two different cytotoxins in *C. pecorum* have different biological functions or host specificity. Related cytotoxins in *E. coli* and *C. difficile* act by glycosylating small GTP-binding proteins of Rho and Ras families, inhibiting the host signalling and regulatory functions [42], lymphocyte activation [43] and by blocking the induction of IFN-γ. Numerous studies have shown the progression of the chlamydial infection cycle to be influenced by IFN-γ production by the host. At low IFN-γ concentrations acute infections typically occur whereas persistence and clearance of infection occurs at medium and high IFN-γ concentrations, respectively [44, 45]. The ability to block IFN-γ production by the host cell may be an important virulence determinant of *C. pecorum* enabling persistent infection of the host with acute disease symptoms occurring when cytotoxins are overexpressed.

Flanking the cytotoxin genes in *C. pecorum* are 4 (PV3056/3) or 5 (P787, W73 and E58) phospholipase D (PLD) genes each containing the conserved HxKx_4_Dx_6_GSxN (HKD) motif essential for the initiation of phosphodiesterase activity and amino acid motifs that are responsible for catalytic activity. PLD genes identified in the plasticity zone of P787, W73 and E58 share 95-99% amino acid sequence identity (CPE2_0554, CPE3_0554, G5S_0938; CPE2_0553, CPE3_0553, G5S_0935; CPE2_0551, CPE3_0551, G5S_0931; CPE2_0550, CPE3_0550, G5S_0930) whereas orthologous PLD genes in PV3056/3 are more divergent (58-71% sequence identity) (CPE1_0553, CPE1_0551, CPE1_0550). The remaining PLD gene is almost identical in E58 and W73 (98% identity, CPE2_0556, G5S_0945) but divergent in the remaining strains (55-79% identity, CPE1_0555, CPE3_0556). The presence of poly(G) and poly(C) homopolymeric tracts ranging in size from 5–19 nucleotides within the PLD genes and the presence of intact variants in the sequence reads of pseudogenes could indicate that these proteins are subject to phase variation by slip-strand pairing [46]. Whilst the function of PLD in *C. pecorum* is currently unknown, PLD can perform numerous functions ranging from DNA hydrolysis, to protein-protein interactions with host signalling pathways, to the more classic lipase function. In *C. trachomatis*, PLD genes located in the PZ have been associated with inclusion formation [47], whereas in other bacteria PLD has been identified as an important virulence determinant involved in dissemination, serum resistance and invasion of epithelial cells [48, 49].

## Conclusions

The complete genome sequence of *C. pecorum* P787, W73 and PV3056/3 was determined by Illumina/Solexa and Roche 454 genome sequencing. Despite the differences in the clinical manifestations of infections caused by the strains, comparative analysis revealed a high level of sequence conservation, gene content and order between the genomes. Additional genomic analyses of strains originating from other non-ruminant host species, such as pig and koala, will determine if the high level of sequence similarity is common to all, or just ruminant strains of *C. pecorum*. In agreement with previous studies [20], differences in the number of clustered tandem repeat sequences in ORF663 were observed between strains isolated from diseased (PV3056/3 and P787) or asymptomatic (W73) animals however, no other genetic differences were observed that may account for the different disease manifestations. A number of metabolic traits were identified in *C. pecorum* that may contribute to its ability to evade the host immune system and enable persistent infections to be established in the host. Specifically, this study has particularly highlighted the absence of genes involved in folate biosynthesis and the presence of tryptophan and biotin biosynthesis pathways. The presence of clustered tandem repeats in surface expressed proteins, 15 polymorphic membrane proteins, two cytotoxin genes and multiple phospholipase D genes that are likely to be subject to phase variable expression may play a role in the invasion of host cells and trigger the switching between persistent and acute disease in the host.

## Methods

### *C. pecorum*strain information, propagation and preparation of gDNA

Three *C. pecorum* strains originating from different geographical regions and disease manifestations were selected for genome sequencing. Strain P787 was isolated in Scotland, in 1977, from the affected synovial fluid of a sheep with polyarthritis. Strain PV3056/3 was isolated in Italy, in 1991, from a cervical swab of a cow with purulent metritis and has subsequently been shown to induce a purulent metritis following inoculation into the uterine body and cervix of cattle [50]. Strain W73 was isolated in Northern Ireland, in 1989, from the faeces of a sheep with an inapparent enteric infection and has subsequently been found to be non-invasive in a mouse model of infection [51].

Strains were propagated in Caco-2 cells grown in RPMI medium supplemented with 5% FBS and 1 μg/ml cyclohexamide. Genomic DNA from PV3056/3 and P787 was derived from the 7th tissue culture passage of original strains propagated in fertile hens’ eggs. W73 was derived from the 6th tissue culture passage of a strain propagated in fertile hens’ eggs, however the passage history prior to this is unknown. Flasks of infected cells were harvested using glass beads followed by centrifugation at 22,000 × *g* for 40 mins. Pellets were washed in ice-cold PBS and re-centrifuged as before. Pellets were resuspended in 20 mM Tris–HCl (pH 7.5)/150 mM KCl/1% sarkosyl and lightly homogenised using a ground glass homogeniser. Homogenised cells were layered onto cushions of 15% sucrose in 20 mM Tris–HCl (pH 7.5)/150 mM KCl/1% sarkosyl and centrifuged at 70,000 × *g* for 45 min at 4°C. Genomic DNA was extracted from pellets using the Wizard DNA extraction kit (Promega).

### Genome sequencing

Genome sequencing was performed by The Gene Pool genomic facility in The University of Edinburgh using Roche 454 GS-FLX and Solexa/Illumina 35-bp paired-end sequencing on standard libraries constructed according to the manufacturers instructions. Reads were assembled using Newbler v2 (Roche) and Velvet v.0.7 [52], combined using minimus2 and mapped to the reference genome of *C. pecorum* E58 [24] to generate 13, 10 and 9 contigs for P787, W73 and PV3056/3 respectively. In total, 12,926,259 (PV3056/3), 8,169,259 (W73) and 10,039,539 (P787) reads obtained from Solexa/Illumina sequencing and 95,683 (PV3056/3), 101,405 (W73) and 65,050 (P787) reads from Roche 454 GS-FLX sequencing were obtained. Following quality filtering, sequencing reads were mapped to the reference genome providing approximately 253× (PV3056/3), 136× (W73) and 59.4× (P787) sequencing coverage. Regions spanning the contig ends were PCR-amplified using Phusion High-fidelity DNA polymerase (NEB) and the sequence determined ensuring that each base was covered by sequence in each direction.

### Sequence annotation and analysis

Protein-encoding genes were predicted using Prodigal [53] and open reading frames (ORFs) consisting of fewer than 30 codons or those overlapping larger open reading frames were eliminated. Frameshifts, point mutations and pseudogenes were corrected or confirmed by visual inspection of mapped reads using Tablet [54]. The origin of replication was determined using Ori-finder [55] and the genomes were adjusted so that the first base was upstream of the hemB gene in the oriC region. Ribosomal RNA genes and tRNA genes were identified using RNAmmer and ARAGORN [56, 57]. Sequences of experimentally validated small non-coding RNAs (sRNA) from chlamydia were downloaded from BSRD [58] and identified in *C. pecorum* genomes using blastn. Functional assignments were made based on homology searches using blastp [59] against protein sequences present in the NCBI nr database and the identification of conserved domains using Pfam [60] and InterProScan protein databases [61]. Signal sequences were predicted using the LipoP 1.0 [62]. KEGG orthology assignments were performed using KAAS [63]. Data collation and annotation was performed using Artemis [64].

Comparative analysis were performed using the following genomes: *C. pecorum* E58 [GenBank: CP002608] [23], *C. abortus* S26/3 [GenBank: CR848038] [25], *C. caviae* GPIC [GenBank: AE015925] [26], *C. felis* Fe/C-56 [GenBank: AP006861] [27], *C. psittaci* 6BC [GenBank: CP002586] [28], *C trachomatis* D/UW-3/CX [GenBank: AE001273] [29]), *C. pneumoniae* AR39 [GenBank: AE002161] and *C. muridarum* Nigg [GenBank: AE002160] [65]. Global genomic comparisons were visualised using ACT [66] with input files generated by the tblastx function in DoubleAct http://www.hpa-bioinfotools.org.uk/pise/double_act.html# with a cutoff score of 0. Comparisons of regions flanking the PZ were performed using default blastn settings in EasyFig [67]. Orthologous gene sets were identified by OrthoMCL-DB using reciprocal blastp with a cutoff of e-5 and 50% match [68]. Genome maps were generated using the CGView Server [69].

### Phylogenetic analyses

Reference sequences were obtained from GenBank and aligned with relevant *C. pecorum* CDSs using MUSCLE [70]. Phylogenetic alignments and tree files are available from the Dryad Digital repository http://doi.org/10.5061/dryad.np597. For ribosomal proteins, 48 individual alignments were concatenated into a single alignment for analysis. For the phylogenetic analysis of cytotoxin genes, GBlocks v 0.91 [71] was used to eliminate regions that could not be unambiguously aligned resulting in 2845 positions being analysed (75% of the original 3766 positions). Phylogenetic analyses were performed using PhyML (for ribosomal proteins and polymorphic membrane proteins) or MrBayes (for cytotoxins) software [72] launched from the TOPALi v2.5package [73] generated using the JTT + G (ribosomal proteins), JTT + I + G (polymorphic membrane proteins) or WAG + I + G (cytotoxins) substitution model that was determined to be the model of best fit based on the BIC criterion. For MrBayes phylogeny, trees were generated using Markov Chain Monte Carlo (MCMC) settings of 2 runs of 625,000 generations with a burn-in of 125,000 generations with trees sampled every 100 runs. For PhyML phylogeny, bootstrap analysis was performed based on 100 replicate trees. Phylogenetic network analysis was performed using SplitsTree [74].

### Nucleotide sequence accession number

Genome sequences of *C. pecorum* strains PV3056/3, W73 and P787 have been deposited in GenBank under the accession numbers CP004033, CP004034, and CP004035, respectively.

## Electronic supplementary material

Additional file 1: Table S1: Location of small regulatory non-coding RNAs (sRNAs) in *C. pecorum* genome sequences. **Table S2**. Identity of pseudogenes in *C. pecorum* genome sequences. **Table S3**. Genes involved in folate biosynthesis in Chlamydiaceae species. **Table S4**. Properties of *C. pecorum* polymorphic membrane (AT domain-containing) proteins. **Table S5**. Type III secretion system structural genes and chaperones identified in *C. pecorum* predicted on the basis of primary sequence similarity (blastp comparison) and domain structure. **Table S6**. Genetic composition of *C. pecorum* plasticity zone. (DOC 146 KB)

Additional file 2: Figure S1: Biotin biosynthesis operon region. Schematic view of the conserved genes dihydropicolinic reductase (*dapB*) and biotin synthase (*bioB*) flanking a variable segment positioned upstream of the biotin biosynthesis operon encoding *bioBFDA*. Dashed lines connect orthologs between the genomes. *C. psittaci* (locus tags G50_0747-G50_0756) and *C. felis* (locus tags CF0294-CF0303) have an identical gene arrangement to *C. abortus. C. pecorum* strains W73, P787 and E58 have an identical arrangement to PV3056/3. **Figure S2**. Phylogenetic network analysis of Pmp autotransporter domains. Phylogenetic network analysis of Pmp autotransporter domains obtained from aligned AT domain protein sequences using NeighborNet analysis performed through the SplitsTree package [72]. **Figure S3**. TMH-family proteins. Schematic view showing regions containing predicted Inc- and TMH-family proteins extending between *pmpD* and *lpxB* in members of the family Chlamydiaceae. Pseudogenes in *C. pecorum* are coloured black. Locus tags are indicated inside each CDS. Dashed lines connect orthologs between genomes. Letters A and B indicate the most closely related TMH protein between *C. pecorum* strains and other chlamydial species. **Figure S4**. Simple sequence repeats (SSR). (A) Schematic view showing conservation of genes surrounding the SSR region and the positioning of corresponding hypothetical proteins or invasin-like genes in other chlamydial species. Dashed lines connect orthologs between the genomes. The SSR region is indicated by the box. (B) Alignment of nucleotide sequences between the 5S rRNA gene and *nqrF* showing the number of repeat sequences (AAAGCACT) in *C. pecorum*. The SSR region is indicated by the box. (PDF 438 KB)

## References

[1] Cavirani S, Cabassi CS, Donofrio G, De Iaco B, Taddei S, Flammini CF (2001). Association between *Chlamydia psittaci* seropositivity and abortion in Italian dairy cows. Prev Vet Med.

[2] DeGraves FJ, Gao D, Hehnen HR, Schlapp T, Kaltenboeck B (2003). Quantitative detection of *Chlamydia psittaci* and *C. pecorum* by high-sensitivity real-time PCR reveals high prevalence of vaginal infection in cattle. J Clin Microbiol.

[3] Jee J, Degraves FJ, Kim T, Kaltenboeck B (2004). High prevalence of natural *Chlamydophila* species infection in calves. J Clin Microbiol.

[4] Lenzko H, Moog U, Henning K, Lederbach R, Diller R, Menge C, Sachse K, Sprague LD (2011). High frequency of chlamydial co-infections in clinically healthy sheep flocks. BMC Vet Res.

[5] Wilson K, Sammin D, Harmeyer S, Nath M, Livingstone M, Longbottom D (2012). Seroprevalence of chlamydial infection in cattle in Ireland. Vet J.

[6] Yousef Mohamad K, Rodolakis A (2010). Recent advances in the understanding of *Chlamydophila pecorum* infections, sixteen years after it was named as the fourth species of the *Chlamydiaceae* family. Vet Res.

[7] Matsumoto A, Manire GP (1970). Electron microscopic observations on the effects of penicillin on the morphology of *Chlamydia psittaci*. J Bacteriol.

[8] Coles AM, Reynolds DJ, Harper A, Devitt A, Pearce JH (1993). Low-nutrient induction of abnormal chlamydial development: a novel component of chlamydial pathogenesis?. FEMS Microbiol Lett.

[9] Raulston JE (1997). Response of *Chlamydia trachomatis* serovar E to iron restriction in vitro and evidence for iron-regulated chlamydial proteins. Infect Immun.

[10] Pantoja LG, Miller RD, Ramirez JA, Molestina RE, Summersgill JT (2001). Characterization of *Chlamydia pneumoniae* persistence in HEp-2 cells treated with gamma interferon. Infect Immun.

[11] Reinhold P, Jaeger J, Liebler-Tenorio E, Berndt A, Bachmann R, Schubert E, Melzer F, Elschner M, Sachse K (2008). Impact of latent infections with *Chlamydophila* species in young cattle. Vet J.

[12] Jaeger J, Liebler-Tenorio E, Kirschvink N, Sachse K, Reinhold P (2007). A clinically silent respiratory infection with *Chlamydophila* spp. in calves is associated with airway obstruction and pulmonary inflammation. Vet Res.

[13] Poudel A, Elsasser TH, Rahman KS, Chowdhury EU, Kaltenboeck B (2012). Asymptomatic endemic *Chlamydia pecorum* infections reduce growth rates in calves by up to 48 percent. PLoS One.

[14] Anderson IE, Baxter SI, Dunbar S, Rae AG, Philips HL, Clarkson MJ, Herring AJ (1996). Analyses of the genomes of chlamydial isolates from ruminants and pigs support the adoption of the new species *Chlamydia pecorum*. Int J Syst Bacteriol.

[15] Jackson M, Giffard P, Timms P (1997). Outer membrane protein A gene sequencing demonstrates the polyphyletic nature of koala *Chlamydia pecorum* isolates. Syst Appl Microbiol.

[16] Kaltenboeck B, Kousoulas KG, Storz J (1993). Structures of and allelic diversity and relationships among the major outer membrane protein (ompA) genes of the four chlamydial species. J Bacteriol.

[17] Fukushi H, Hirai K (1989). Genetic diversity of avian and mammalian *Chlamydia psittaci* strains and relation to host origin. J Bacteriol.

[18] Salinas J, Souriau A, De Sa C, Andersen AA, Rodolakis A (1996). Serotype 2-specific antigens from ruminant strains of *Chlamydia pecorum* detected by monoclonal antibodies. Comp Immunol Microbiol Infect Dis.

[19] Liu Z, Rank R, Kaltenboeck B, Magnino S, Dean D, Burall L, Plaut RD, Read TD, Myers G, Bavoil PM (2007). Genomic plasticity of the rrn-nqrF intergenic segment in the Chlamydiaceae. J Bacteriol.

[20] Yousef Mohamad K, Roche SM, Myers G, Bavoil PM, Laroucau K, Magnino S, Laurent S, Rasschaert D, Rodolakis A (2008). Preliminary phylogenetic identification of virulent *Chlamydophila pecorum* strains. Infect Genet Evol.

[21] Yousef Mohamad K, Rekiki A, Myers G, Bavoil PM, Rodolakis A (2008). Identification and characterisation of coding tandem repeat variants in incA gene of *Chlamydophila pecorum*. Vet Res.

[22] Jelocnik M, Frentiu FD, Timms P, Polkinghorne A (2013). Multilocus sequence analysis provides insights into molecular epidemiology of *Chlamydia pecorum* infections in Australian sheep, cattle and koalas. J Clin Microbiol.

[23] Mojica S, Huot Creasy H, Daugherty S, Read TD, Kim T, Kaltenboeck B, Bavoil P, Myers GS (2011). Genome sequence of the obligate intracellular animal pathogen *Chlamydia pecorum* E58. J Bacteriol.

[24] Pannekoek Y, Dickx V, Beeckman DSA, Jolley KA, Keijzers WC, Vretou E, Maiden MCJ, Vanrompay D, van der Ende A (2010). Multi locus sequence typing of *Chlamydia* reveals an association between *Chlamydia psittaci* genotypes and host species. Plos one.

[25] Thomson NR, Yeats C, Bell K, Holden MT, Bentley SD, Livingstone M, Cerdeño-Tárraga AM, Harris B, Doggett J, Ormond D, Mungall K, Clarke K, Feltwell T, Hance Z, Sanders M, Quail MA, Price C, Barrell BG, Parkhill J, Longbottom D (2005). The *Chlamydophila abortus* genome sequence reveals an array of variable proteins that contribute to interspecies variation. Genome Res.

[26] Read TD, Myers GSA, Brunham RC, Nelson WC, Paulsen IT, Heidelberg J, Holtzapple E, Khouri H, Federova NB, Carty HA, Umayam LA, Haft DH, Peterson J, Beanan MJ, White O, Salzberg SL, Hsia R-C, McClarty G, Rank RG, Bavoil PM, Fraser CM (2003). Genome sequence of *Chlamydophila caviae* (*Chlamydia psittaci* GPIC): examining the role of niche-specific genes in the evolution of the Chlamydiaceae. Nucleic Acids Res.

[27] Azuma Y, Hirakawa H, Yamashita A, Cai Y, Rahman MA, Suzuki H, Mitaku S, Toh H, Goto S, Murakami T, Sugi K, Hayashi H, Fukushi H, Hattori M, Kuhara S, Shirai M (2006). Genome sequence of the cat pathogen, *Chlamydophila felis*. DNA Res.

[28] Grinblat-Huse V, Drabek EF, Creasy HH, Daugherty SC, Jones KM, Santana-Cruz I, Tallon LJ, Read TD, Hatch TP, Bavoil P, Myers GS (2011). Genome sequences of the zoonotic pathogens *Chlamydia psittaci* 6BC and Cal10. J Bacteriol.

[29] Stephens RS, Kalman S, Lammel C, Fan J, Marathe R, Aravind L, Mitchell W, Olinger L, Tatusov RL, Zhao Q, Koonin EV, Davis RW (1998). Genome sequence of an obligate intracellular pathogen of humans: *Chlamydia trachomatis*. Science.

[30] Taylor MW, Feng GS (1991). Relationship between interferon-γ, indoleamine 2,3-dioxygenase, and tryptophan catabolism. FASEB J.

[31] Beatty WI, Belanger TA, Desai AA, Morrison RP, Byrne GI (1994). Tryptophan depletion as a mechanism of gamma interferon-mediated chlamydial persistence. Infect Immun.

[32] Eudes A, Erkens GB, Slotboom DJ, Rodionov DA, Naponelli V, Hanson AD (2008). Identification of genes encoding the folate- and thiamine-binding membrane proteins in Firmicutes. J Bacteriol.

[33] Myllykallio H, Leduc D, Filee J, Liebl U (2003). Life without dihydrofolate reductase FolA. Trends Microbiol.

[34] Henderson IR, Lam AC (2001). Polymorphic proteins of *Chlamydia* spp. – autotransporters beyond the Proteobacteria. Trends Microbiol.

[35] Siboo IR, Chambers HF, Sullam PM (2005). Role of SraP, a serine-rich surface protein of *Staphylococcus aureus*, in binding to human platelets. Infect Immun.

[36] Seifert KN, Adderson EE, Whiting AA, Bohnsack JF, Crowley PJ, Brady LJ (2006). A unique serine-rich repeat protein (Srr-2) and novel surface antigen (epsilon) associated with a virulent lineage of serotype III *Stretococcus agalactiae*. Microbiol.

[37] Gravekamp C, Rosner B, Madoff LC (1998). Deletion of repeats in the alpha C protein enhances the pathogenicity of group B streptococci in immune mice. Infect Immun.

[38] Rockey DD, Grosenbach D, Hruby DE, Peacock MG, Helnzen RA, Hackstadt T (1997). *Chlamydia psittaci* IncA is phosphorylated by the host cell and exposed on the cytoplasmic face of the developing inclusion. Mol Microbiol.

[39] Yousef Mohamad K, Rekiki A, Berri M, Rodolakis A (2010). Recombinant 35-kDa inclusion membrane protein IncA as a candidate antigen for serodiagnosis of *Chlamydophila pecorum*. Vet Microbiol.

[40] Hackstadt T, Scidmore-Carlson MA, Shaw EI, Fisher ER (1999). The *Chlamydia trachomatis* IncA protein is required for homotypic vesicle fusion. Cell Microbiol.

[41] Geisler WM, Suchland RJ, Rockey DD, Stamm WE (2001). Epidemiology and clinical manifestations of unique *Chlamydia trachomatis* isolates that occupy nonfusogenic inclusions. J Infect Dis.

[42] Von Eichel-Streiber C, Boquet P, Sauerborn M, Thelestam M (1996). Large clostridial cytotoxins-a family of glycosyltransferases modifying small GTP-binding proteins. Trends Microbiol.

[43] Klapproth JA, Scaletsky ICA, McNamara BP, Lai L, Malstrom C, James SP, Donnenberg MS (2000). A large toxin from pathogenic *Escherichia coli* strains that inhibits lymphocyte activation. Infect Immun.

[44] Entrican G, Brown J, Graham S (1998). Cytokines and the protective host immune response to *Chlamydia psittaci*. Comp Immun Microbiol Infect Dis.

[45] Shemer Y, Sarov I (1985). Inhibition of growth of *Chlamydia trachomatis* by human gamma interferon. Infect Immun.

[46] Viratyosin W, Campbell LA, Kuo CC, Rockey DD (2002). Intrastrain and interstrain genetic variation within a paralogous gene family in *Chlamydia pneumoniae*. BMC Microbiol.

[47] Nelson DE, Crane DD, Taylor LD, Dorward DW, Goheen MM, Caldwell HD (2006). Inhibition of chlamydiae by primary alcohols correlates with the strain-specific complement of plasticity zone phospholipase D genes. Infect Immun.

[48] Jacobs AC, Hood I, Boyd KL, Olson PD, Morrison JM, Carson S, Sayood K, Iwen PC, Skaar EP, Dunman PM (2010). Inactivation of phospholipase D diminishes *Acinetobacter baumannii* pathogenesis. Infect Immun.

[49] Edwards JL, Entz DD, Apicella MA (2003). Gonococcal phospholipase D modulates the expression and function of complement receptor 3 in primary cervical epithelial cells. Infect Immun.

[50] Jones GE, Machell DA, Biolatti B, Appino S, Stevens RS, Byrne GI, Christianson G (1998). Experimental infections of the genital tract of cattle with *Chlamydia psittaci* and *Chlamydia pecorum*. Proceedings of the Ninth International Symposium on Human Chlamydial Infection.

[51] Denamur E, Sayada C, Souriau A, Orfila J, Rodolakis A, Elion J (1991). Restriction pattern of the major outer-membrane protein gene provides evidence for a homogeneous invasive group among ruminant isolates of *Chlamydia psittaci*. J Gen Microbiol.

[52] Zerbino DR, Birney E (2008). Velvet: algorithms for de novo short read assembly using de Bruijn graphs. Genome Res.

[53] Hyatt D, Chen GL, Locascio PF, Land ML, Larimer FW, Hauser LJ (2010). Prodigal: prokaryotic gene recognition and translation initiation site identification. BMC Bioinforma.

[54] Milne I, Stephen G, Bayer M, Cock PJ, Pritchard L, Cardle L, Shaw P, Marshall D (2013). Using tablet for visual exploration of second-generation sequencing data. Brief Bioinform.

[55] Gao F, Zhang CT (2008). Ori-Finder: a web-based system for finding oriCs in unannotated bacterial genomes. BMC Bioinforma.

[56] Lagesen K, Hallin P, Rødland EA, Staerfeldt HH, Rognes T, Ussery DW (2007). RNAmmer: consistent and rapid annotation of ribosomal RNA genes. Nucleic Acids Res.

[57] Laslett D, Canback B (2004). ARAGORN, a program to detect tRNA genes and tmRNA genes in nucleotide sequences. Nucleic Acids Res.

[58] Li L, Huang D, Cheung MK, Nong W, Huang Q, Kwan HS (2013). BSRD: a repository for bacterial small regulatory RNA. Nucleic Acids Res.

[59] Atschul SF, Gish W, Miller W, Myers EW, Lipman DJ (1990). Basic local alignment search tool. J Mol Biol.

[60] Finn RD, Tate J, Mistry J, Coggill PC, Sammut JS, Hotz HR, Ceric G, Forslund K, Eddy SR, Sonnhammer EL, Bateman A (2008). The Pfam protein families database. Nucleic Acids Res Database Issue.

[61] Zdobnov EM, Apweiler R (2001). InterProScan - an integration platform for the signature-recognition methods in InterPro. Bioinformatics.

[62] Juncker AS, Willenbrock H, von Heijne G, Nielsen H, Brunak S, Krogh A (2003). Prediction of lipoprotein signal peptides in Gram-negative bacteria. Protein Sci.

[63] Moriya Y, Itoh M, Okuda S, Yoshizawa A, Kanehisa M (2007). KAAS: an automatic genome annotation and pathway reconstruction server. Nucleic Acids Res.

[64] Rutherford K, Parkhill J, Crook J, Horsnell T, Rice P, Rajandream MA, Barrell B (2000). Artemis: sequence visualization and annotation. Bioinformatics.

[65] Read TD, Brunham RC, Shen C, Gill SR, Heidelberg JF, White O, Hickey EK, Peterson J, Utterback T, Berry K, Bass S, Linher K, Weidman J, Khouri H, Craven B, Bowman C, Dodson R, Gwinn M, Nelson W, DeBoy R, Kolonay J, McClarty G, Salzberg SL, Eisen J, Fraser CM (2000). Genome sequences of *Chlamydia trachomatis* MoPn and *Chlamydia pneumoniae* AR39. Nucleic Acids Res.

[66] Carver TJ, Rutherford KM, Berriman M, Rajandream MA, Barrell BG, Parkhill J (2005). ACT: the Artemis comparison tool. Bioinformatics.

[67] Sullivan MJ, Petty NK, Beatson SA (2011). Easyfig: a genome comparison visualiser. Bioinformatics.

[68] Chen F, Mackey AJ, Stoeckert CJ, Roos DS (2006). OrthoMCL-DB: querying a comprehensive multi-species collection of ortholog groups. Nucleic Acids Res.

[69] Grant JR, Stothard P (2008). The CGView server: a comparative genomics tool for circular genomes. Nucleic Acids Res.

[70] Edgar RC (2004). MUSCLE: multiple sequence alignment with high accuracy and high throughput. Nucleic Acids Res.

[71] Castresana J (2000). Selection of conserved blocks from multiple alignments for their use in phylogenetic analysis. Mol Biol Evol.

[72] Ronquist F, Huelsenbeck JP (2003). MrBayes 3: Bayesian phylogenetic inference under mixed models. Bioinformatics.

[73] Milne I, Lindner D, Bayer M, Husmeier D, McGuire G, Marshall DF, Wright F (2009). TOPALi v2: a rich graphical interface for evolutionary analyses of multiple alignments on HPC clusters and multi-core desktops. Bioinformatics.

[74] Huson DH, Bryant D (2006). Application of phylogenetic networks in evolutionary studies. Mol Biol Evol.

