# Supplementary material for: Genome sequencing and comparative analysis of three Chlamydia pecorum strains associated with different pathogenic outcomes
Source: BMC Genomics. 2014 Jan 14;15(1):23. doi: 10.1186/1471-2164-15-23 (PMC3932018; doi:10.1186/1471-2164-15-23)
Supplement: Supplementary file 1 — Additional file 1: Table S1: Location of small regulatory non-coding RNAs (sRNAs) in C. pecorum genome sequences. Table S2. Identity of pseudogenes in C. pecorum genome sequences. Table S3. Genes involved in folate biosynthesis in Chlamydiaceae species. Table S4. Properties of C. pecorum polymorphic membrane (AT domain-containing) proteins. Table S5. Type III secretion system structural genes and chaperones identified in C. pecorum predicted on the basis of primary sequence similarity (blastp comparison) and domain structure. Table S6. Genetic composition of C. pecorum plasticity zone. (DOC 146 KB) [file 12864_2013_7002_MOESM1_ESM.doc]

Supplementary Table 1. Location of small regulatory non-coding RNAs (sRNAs) in *C. pecorum* genome sequences.

| Strain | sRNA | | |
| --- | --- | --- | --- |
|  | ssrA | RNaseP | ffs |
| PV3056/3 | 715240-715663 | 227644-227238 | 423836-423938 |
| W73 | 717171-717594 | 227598-227192 | 424191-424293 |
| P787 | 717028-717451 | 227549-227143 | 423757-423859 |
| E58 | 1022853-1023279 | 533599-533196 | 729819-729921 |

Supplementary Table 2. Identity of pseudogenes in *C. pecorum* genome sequences.

| Strain | Locus tag | Protein description | Type of change |
| --- | --- | --- | --- |
| PV3056/3 | CPE1_0551 | Phospholipase D family protein | Frameshift poly G (indicative of phase variation) |
|  | CPE1_0553 | Phospholipase D family protein | Frameshift poly G (indicative of phase variation) AND frameshift mutation |
|  | CPE1_0556 | Hypothetical protein | Frameshift mutation |
|  | CPE1_0577 | Peptide ABC transporter, permease protein | Frameshift mutation poly A |
|  | CPE1_0724 | Hypothetical protein | Frameshift mutation |
|  | CPE1_0764 | Hypothetical protein | Frameshift mutation poly A |
| W73 | CPE2_0550 | Phospholipase D family protein | Frameshift poly C (indicative of phase variation) |
|  | CPE2_0551 | Phospholipase D family protein | Frameshift poly G (indicative of phase variation) |
|  | CPE2_0556 | Phospholipase D family protein | Frameshift poly G (indicative of phase variation) |
| P787 | CPE3_0551 | Phospholipase D family protein | Frameshift poly G (indicative of phase variation) |
|  | CPE3_0557 | Hypothetical protein | Frameshift poly G (indicative of phase variation) |
|  | CPE3_0810 | Hypothetical protein | Frameshift poly T (indicative of phase variation) |
| E58 | G5S_0527 | Polymorphic membrane protein A family | Frameshift |

Supplementary Table 3. Genes involved in folate biosynthesis in Chlamydiaceae species.

|  |  |  |  | Gene locus |  |  |  |  |  |  |  |
| --- | --- | --- | --- | --- | --- | --- | --- | --- | --- | --- | --- |
| Species | Strain | Accession No. |  | *fmt* | *glyA* | *folD* | *ygfA* | *folA* | *folKP* | *folB (folX)* | *thyX (thy1)* |
| *C. pecorum* | PV3056/3 | CP004033 |  | CPE1_0094 | CPE1_0229 | CPE1_0433 | CPE1_0955 | - | - | - | CPE1_0968 |
| *C. pecorum* | W73 | CP004034 |  | CPE2_0094 | CPE2_0229 | CPE2_0433 | CPE2_0956 | - | - | - | CPE2_0969 |
| *C. pecorum* | P787 | CP004035 |  | CPE3_0094 | CPE3_0229 | CPE3_0433 | CPE3_0956 | - | - | - | CPE3_0969 |
| *C. pecorum* | E58 | CP002608 |  | G5S_0396 | G5S_0551 | G5S_0798 | G5S_0281 | - | - | - | G5S_0296 |
| *C. abortus* | S26/3 | CR848038 |  | CAB091 | CAB220 | CAB434 | CAB964 | CAB968 | CAB969 | CAB970 | CAB981 |
| *C. psittaci* | 6BC | CP002549 |  | CPSIT_0103 | CPSIT_0250 | CPSIT_0487 | CPSIT_1054 | CPSIT_1058 | CPSIT_1059 | CPSIT_1060 | CPSIT_1072 |
| *C. felis* | Fe/C-56 | AP006861 |  | CF0915 | CF0782 | CF0559 | CF0019 | CF0015 | CF0014 | CF0013 | CF0001 |
| *C. caviae* | GPIC | AE015925 |  | CCA00091 | CCA00224 | CCA00448 | CCA00994 | CCA00998 | CCA00999 | CCA01000 | CCA01011 |
| *C. pneumoniae* | CWL029 | AE001363 |  | CPn0649 | CPn0521 | CPn0335 | CPn0763 | CPn0759 | CPn0758 | CPn0757 | CPn0746 |
| *C. muridarum* | Nigg | AE002160 |  | TC_0817 | TC_0716 | TC_0350 | TC_0018 | TC_0902 | TC_0903 | TC_0904 | TC_0921 |
| *C. trachomatis* | D/UW-3/CX | AE001273 |  | CT530 | CT432 | CT078 | CT649 | CT612 | CT613 | CT614 | CT632 |

Supplementary Table 4. Properties of *C. pecorum* Polymorphic membrane (AT domain-containing) proteins.

|  | Locus tag | Pmp family | No. aa | Mw^a^ | pI^a^ | Signal peptidase | Cleavage site | Conserved domains |
| --- | --- | --- | --- | --- | --- | --- | --- | --- |
| PV3056/3 | CPE1_0210 | B | 1698 | 179.2 | 5.94 | type I | ITAFG|EPNSV | POMP repeat, AT beta-domain, PMP_M, Pectin lyase fold, Parallel beta-helix repeat |
|  | CPE1_0211 | A | 947 | 102.6 | 8.72 | type I | SYLLA\|EDELL | POMP repeat, AT beta-domain, PMP_M |
|  | CPE1_0275 | E | 938 | 102.5 | 6.84 | type I |  | POMP repeat, AT beta-domain, PMP_M |
|  | CPE1_0276 | E | 974 | 106.8 | 7.15 | - | - | POMP repeat, AT beta-domain, PMP_M |
|  | CPE1_0277 | H | 1006 | 107.4 | 6.45 | type I | GASWS\|FGSHH | POMP repeat, AT beta-domain, PMP_M |
|  | CPE1_0278 | G | 1009 | 106.0 | 6.78 | type I | SSVFA\|ETIPI | POMP repeat, AT beta-domain, PMP_M |
|  | CPE1_0281 | G | 956 | 104.0 | 6.83 | - | - | POMP repeat, AT beta-domain, PMP_M, Pectin lyase fold |
|  | CPE1_0282 | G | 819 | 89.7 | 6.16 | type I | SYSLS\|ASVIY | POMP repeat, AT beta-domain, PMP_M |
|  | CPE1_0283 | G | 1062 | 110.8 | 6.65 | type I | PSLCA\|TETPL | POMP repeat, AT beta-domain, PMP_M |
|  | CPE1_0284 | G | 889 | 94.3 | 5.20 | type I | SLVSA\|ETPLG | POMP repeat, AT beta-domain, PMP_M, Pectin lyase fold |
|  | CPE1_0285 | G | 935 | 98.7 | 6.34 | type I | FSLSA\|ATTNL | POMP repeat, AT beta-domain, PMP_M |
|  | CPE1_0286 | G | 958 | 101.0 | 6.23 | type I | FSVYA\|ANTNL | POMP repeat, AT beta-domain, PMP_M |
|  | CPE1_0287 | G | 1397 | 146.3 | 4.88 | type I | TETNS\|EPDQN | POMP repeat, AT beta-domain, PMP_M, Parallel beta-helix repeat |
|  | CPE1_0679 | G | 947 | 99.3 | 6.13 | type I | SIAFA\|APDQV | POMP repeat, AT beta-domain, PMP_M |
|  | CPE1_0766 | D | 1376 | 147.0 | 5.01 | - | - | POMP repeat, AT beta-domain, PMP_M |
| W73 | CPE2_0210 | B | 1687 | 177.6 | 5.99 | type I | ITAFG|EPNSV | POMP repeat, AT beta-domain, PMP_M, Pectin lyase fold, Parallel beta-helix repeat |
|  | CPE2_0211 | A | 947 | 102.6 | 8.91 | type I | SYLLA\|EDELL | POMP repeat, AT beta-domain, PMP_M |
|  | CPE2_0275 | E | 955 | 104.6 | 8.66 | type I | PSLYA\|TNTPL | POMP repeat, AT beta-domain, PMP_M |
|  | CPE2_0276 | E | 977 | 106.8 | 7.12 | - | - | POMP repeat, AT beta-domain, PMP_M |
|  | CPE2_0277 | H | 1003 | 106.8 | 7.25 | type I | FGSHH\|NQNVA | POMP repeat, AT beta-domain, PMP_M |
|  | CPE2_0278 | G | 1008 | 105.8 | 8.68 | type I | SPVFA\|ETIPI | POMP repeat, AT beta-domain, PMP_M |
|  | CPE2_0281 | G | 956 | 104.1 | 6.83 | - | - | POMP repeat, AT beta-domain, PMP_M, Pectin lyase fold |
|  | CPE2_0282 | G | 819 | 89.7 | 6.23 | type I | SYSLS\|ASVIY | POMP repeat, AT beta-domain, PMP_M |
|  | CPE2_0283 | G | 1064 | 110.9 | 6.17 | type I | PSLCA\|TETLL | POMP repeat, AT beta-domain, PMP_M |
|  | CPE2_0284 | G | 888 | 93.9 | 5.03 | type I | FSLFS\|ADTPL | POMP repeat, AT beta-domain, PMP_M |
|  | CPE2_0285 | G | 938 | 98.8 | 6.16 | type I | FSLSA\|ATTNL | POMP repeat, AT beta-domain, PMP_M |
|  | CPE2_0286 | G | 950 | 99.7 | 5.66 | type I | FSVYA\|ANVDL | POMP repeat, AT beta-domain, PMP_M |
|  | CPE2_0287 | G | 1417 | 148.6 | 4.62 | type I | TETQP\|ADETK | POMP repeat, AT beta-domain, PMP_M |
|  | CPE2_0680 | G | 946 | 99.1 | 6.41 | type I | SIAFA\|APDQV | POMP repeat, AT beta-domain, PMP_M |
|  | CPE2_0767 | D | 1376 | 146.9 | 4.97 | - | - | POMP repeat, AT beta-domain, PMP_M |
| P787 | CPE3_0210 | B | 1677 | 176.8 | 6.01 | type I | ITAFG|EPNSV | POMP repeat, AT beta-domain, PMP_M, Pectin lyase fold, Parallel beta-helix repeat |
|  | CPE3_0211 | A | 947 | 102.6 | 8.93 | type I | SYLLA\|EDELL | POMP repeat, AT beta-domain, PMP_M |
|  | CPE3_0275 | E | 955 | 104.6 | 8.66 | type I | PSLYA\|TNTPL | POMP repeat, AT beta-domain, PMP_M, |
|  | CPE3_0276 | E | 977 | 106.8 | 7.12 | - |  | POMP repeat, AT beta-domain, PMP_M |
|  | CPE3_0277 | H | 1003 | 106.9 | 7.25 | type I | FGSHH\|NQNVA | POMP repeat, AT beta-domain, PMP_M |
|  | CPE3_0278 | G | 1016 | 106.7 | 8.68 | type I | SPVFA\|KIVPI | POMP repeat, AT beta-domain, PMP_M |
|  | CPE3_0281 | G | 956 | 104.1 | 6.69 | - |  | POMP repeat, AT beta-domain, PMP_M, Pectin lyase fold |
|  | CPE3_0282 | G | 819 | 89.8 | 6.25 | type I | SYSLS\|ASVIY | POMP repeat, AT beta-domain, PMP_M |
|  | CPE3_0283 | G | 1063 | 110.9 | 6.38 | type I | PSLCA\|TETPL | POMP repeat, AT beta-domain, PMP_M |
|  | CPE3_0284 | G | 886 | 93.8 | 5.05 | type I | SLVSA\|ETPLG | POMP repeat, AT beta-domain, PMP_M |
|  | CPE3_0285 | G | 937 | 98.8 | 6.23 | type I | FSLSA\|ATTNL | POMP repeat, AT beta-domain, PMP_M |
|  | CPE3_0286 | G | 948 | 99.8 | 5.72 | type I | FSVYA\|ANVDL | POMP repeat, AT beta-domain, PMP_M |
|  | CPE3_0287 | G | 1465 | 153.3 | 4.56 | type I | TETQP\|ADETK | POMP repeat, AT beta-domain, PMP_M |
|  | CPE3_0680 | G | 946 | 99.2 | 6.26 | type I | SIAFA\|APDQV | POMP repeat, AT beta-domain, PMP_M |
|  | CPE3_0767 | D | 1376 | 147.0 | 4.96 | - |  | POMP repeat, AT beta-domain, PMP_M |

Supplementary Table 5. Type III secretion system structural genes and chaperones identified in *C. pecorum* predicted on the basis of primary sequence similarity (BLASTP comparison) and domain structure.

| Locus Tag | | | Description | Required for secretion? |
| --- | --- | --- | --- | --- |
| PV3056/3 | W73 | P787 |  |  |
| CPE1_0031 | CPE2_0031 | CPE3_0031 | Tir chaperone protein (CesT) family | No |
| CPE1_0033 | CPE2_0033 | CPE3_0033 | Putative needle chaperone protein SctE | Yes |
| CPE1_0034 | CPE2_0034 | CPE3_0034 | Putative Type III secretion system needle protein SctF | Yes |
| CPE1_0035 | CPE2_0035 | CPE3_0035 | Putative Type III secretion system chaperone SctG | No |
| CPE1_0037 | CPE2_0037 | CPE3_0037 | Type III secretion system ATPase SctN | Yes |
| CPE1_0040 | CPE2_0040 | CPE3_0040 | Putative type III secretion system apparatus protein SctQ | Yes |
| CPE1_0042 | CPE2_0042 | CPE3_0042 | Type III secretion protein SctC | Yes |
| CPE1_0413 | CPE2_0413 | CPE3_0413 | Type III secretion system protein | No |
| CPE1_0442 | CPE2_0442 | CPE3_0442 | Tir chaperone protein (CesT) family | No |
| CPE1_0443 | CPE2_0443 | CPE3_0443 | Type III secretion system protein SctW | Yes |
| CPE1_0444 | CPE2_0444 | CPE3_0444 | Type III secretion inner membrane protein SctV | Yes |
| CPE1_0445 | CPE2_0445 | CPE3_0445 | Type III secretion inner membrane protein SctU | Yes |
| CPE1_0894 | CPE2_0895 | CPE3_0895 | Type III secretion system membrane protein SctJ | Yes |
| CPE1_0896 | CPE2_0897 | CPE3_0897 | Type III secretion apparatus protein SctL | Yes |
| CPE1_0897 | CPE2_0898 | CPE3_0898 | Type III secretion apparatus protein SctR | Yes |
| CPE1_0898 | CPE2_0899 | CPE3_0899 | Type III secretion inner membrane protein SctS | Yes |
| CPE1_0899 | CPE2_0900 | CPE3_0900 | Type III secretion inner membrane protein SctT | Yes |
| CPE1_0911 | CPE2_0912 | CPE3_0912 | Type III secretion system chaperone SycD/LcrH | No |

Supplementary Table 6. Genetic composition of *C. pecorum* plasticity zone

| Strain | | | | Product | Pseudogene |
| --- | --- | --- | --- | --- | --- |
| PV3056/3 | W73 | P787 | E58 |  |  |
| CPE1_0546 | CPE2_0546 | CPE3_0546 | G5S_0926 | Inosine-5’-monophosphate dehydrogenase |  |
| CPE1_0547 | CPE2_0547 | CPE3_0547 | G5S_0927 | GMP synthase |  |
| CPE1_0548 | CPE2_0548 | CPE3_0548 | G5S_0928 | Adenosine/AMP deaminase superfamily |  |
| CPE1_0549 | CPE2_0549 | CPE3_0549 | G5S_0929 | MAC/perforin domain protein |  |
| CPE1_0550 | CPE2_0550 | CPE3_0550 | G5S_0930 | Phospholipase D family protein | W73 |
| CPE1_0551 | CPE2_0551 | CPE3_0551 | G5S_0931 | Phospholipase D family protein | PV3056/3, W73, P787 |
| CPE1_0552 | CPE2_0552 | CPE3_0552 | G5S_0934 | Glycosyltransferase, DXD sugar-binding domain containing protein |  |
|  | CPE2_0553 | CPE3_0553 | G5S_0935 | Phospholipase D family protein |  |
| CPE1_0553 | CPE2_0554 | CPE3_0554 | G5S_0938 | Phospholipase D family protein | PV3056/3, E58 (not annotated) |
| CPE1_0554 | CPE2_0555 | CPE3_0555 | G5S_0942 | Glycosyltransferase, DXD sugar-binding domain containing protein |  |
| CPE1_0555 | CPE2_0556 | CPE3_0556 | G5S_0945 | Phospholipase D family protein | W73 |
| CPE1_0556 | CPE2_0557 | CPE3_0557 | not annotated | Hypothetical protein | PV3056/3, P787 |
| CPE1_0557 | CPE2_0558 | CPE3_0558 | G5S_0948 | Hypothetical protein |  |
| CPE1_0558 | CPE2_0559 | CPE3_0559 | G5S_0949 | Hypothetical protein |  |
| CPE1_0559 | CPE2_0560 | CPE3_0560 | G5S_0950 | Hypothetical protein |  |
| CPE1_0560 | CPE2_0561 | CPE3_0561 | G5S_0951 | Acetyl-CoA carboxylase, biotin carboxylase subunit |  |
| CPE1_0561 | CPE2_0562 | CPE3_0562 | G5S_0952 | Acetyl-CoA carboxylase, biotin carboxyl carrier protein subunit |  |
