# Supplementary material for: Genome sequencing and comparative analysis of three Chlamydia pecorum strains associated with different pathogenic outcomes
Source: BMC Genomics. 2014 Jan 14;15(1):23. doi: 10.1186/1471-2164-15-23 (PMC3932018; doi:10.1186/1471-2164-15-23)
Supplement: Supplementary file 2 — Additional file 2: Figure S1: Biotin biosynthesis operon region. Schematic view of the conserved genes dihydropicolinic reductase (dapB) and biotin synthase (bioB) flanking a variable segment positioned upstream of the biotin biosynthesis operon encoding bioBFDA. Dashed lines connect orthologs between the genomes. C. psittaci (locus tags G50_0747-G50_0756) and C. felis (locus tags CF0294-CF0303) have an identical gene arrangement to C. abortus. C. pecorum strains W73, P787 and E58 have an identical arrangement to PV3056/3. Figure S2. Phylogenetic network analysis of Pmp autotransporter domains. Phylogenetic network analysis of Pmp autotransporter domains obtained from aligned AT domain protein sequences using NeighborNet analysis performed through the SplitsTree package [72]. Figure S3. TMH-family proteins. Schematic view showing regions containing predicted Inc- and TMH-family proteins extending between pmpD and lpxB in members of the family Chlamydiaceae. Pseudogenes in C. pecorum are coloured black. Locus tags are indicated inside each CDS. Dashed lines connect orthologs between genomes. Letters A and B indicate the most closely related TMH protein between C. pecorum strains and other chlamydial species. Figure S4. Simple sequence repeats (SSR). (A) Schematic view showing conservation of genes surrounding the SSR region and the positioning of corresponding hypothetical proteins or invasin-like genes in other chlamydial species. Dashed lines connect orthologs between the genomes. The SSR region is indicated by the box. (B) Alignment of nucleotide sequences between the 5S rRNA gene and nqrF showing the number of repeat sequences (AAAGCACT) in C. pecorum. The SSR region is indicated by the box. (PDF 438 KB) [file 12864_2013_7002_MOESM2_ESM.pdf]

Supplementary Figure 1

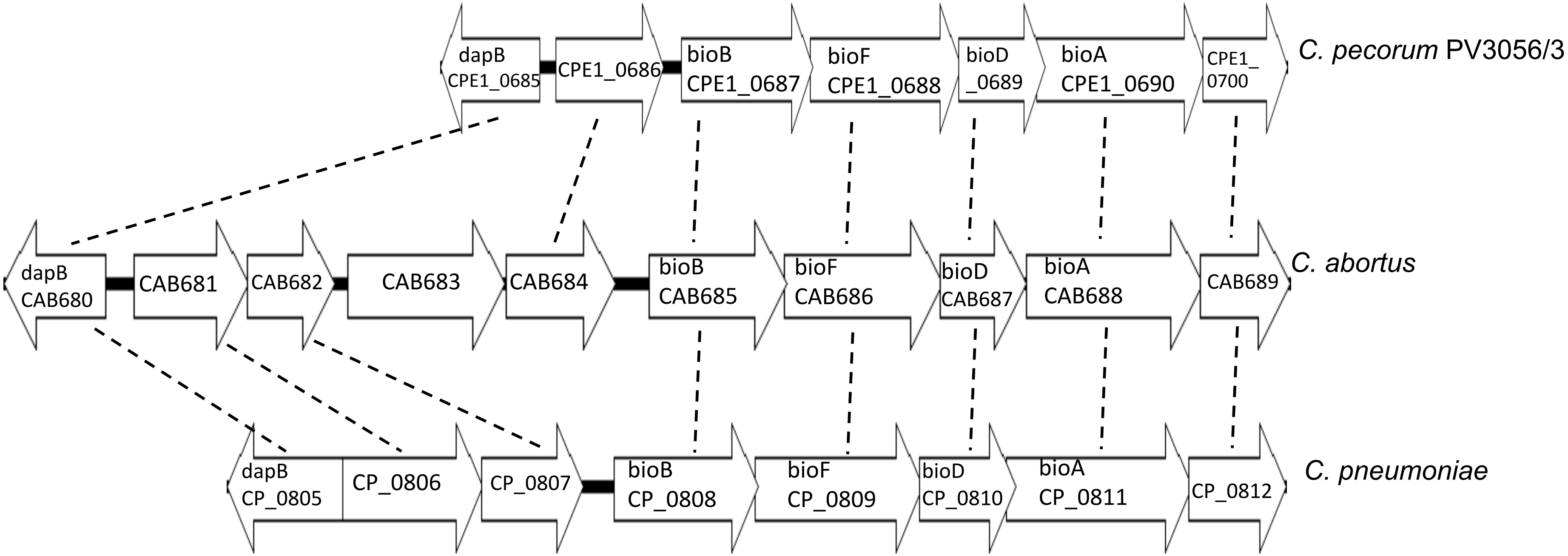

Supplementary Figure 2

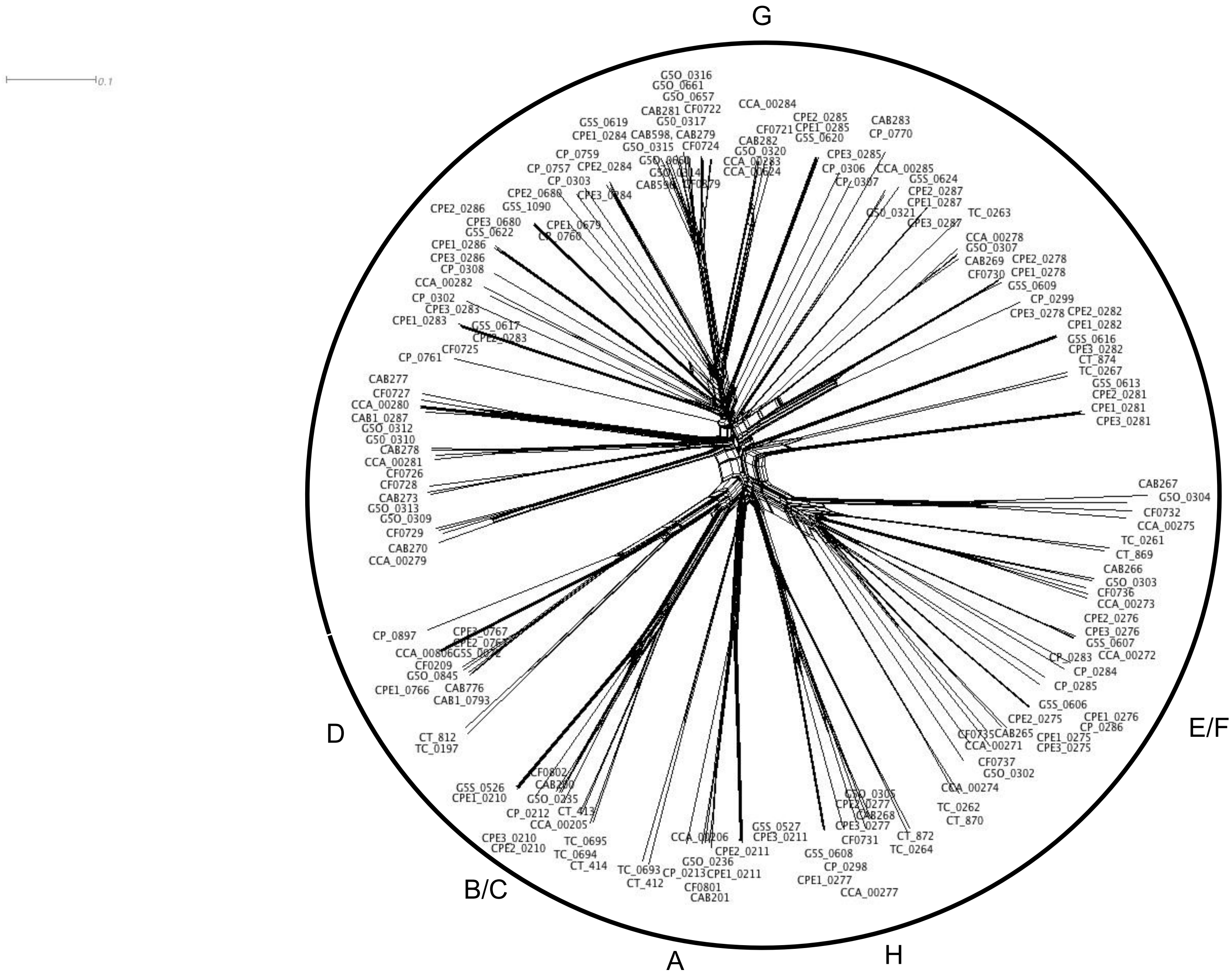

Phi test for Recombination P = 0.02173

Supplementary Figure 3

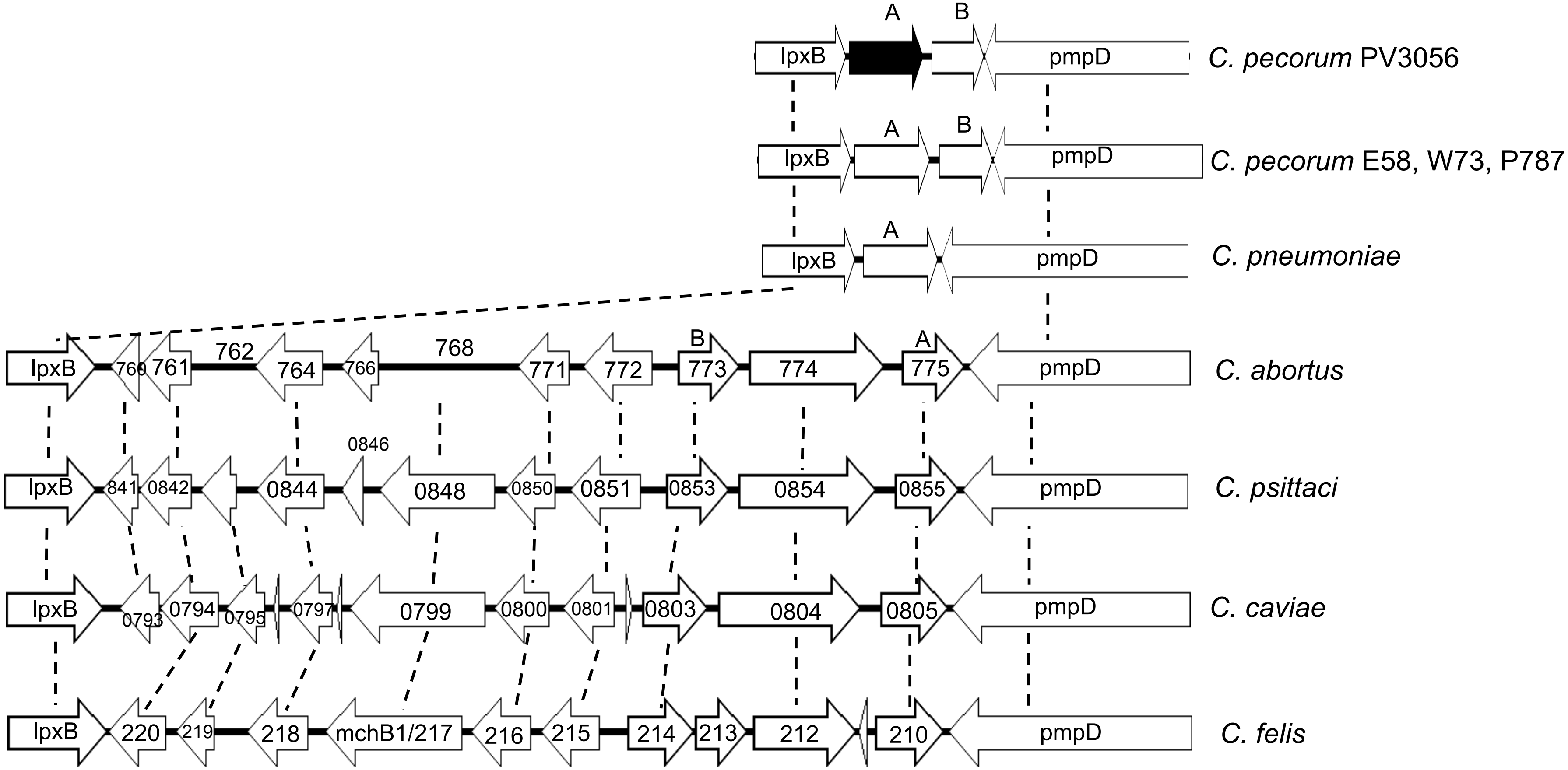

Supplementary Figure 4

A

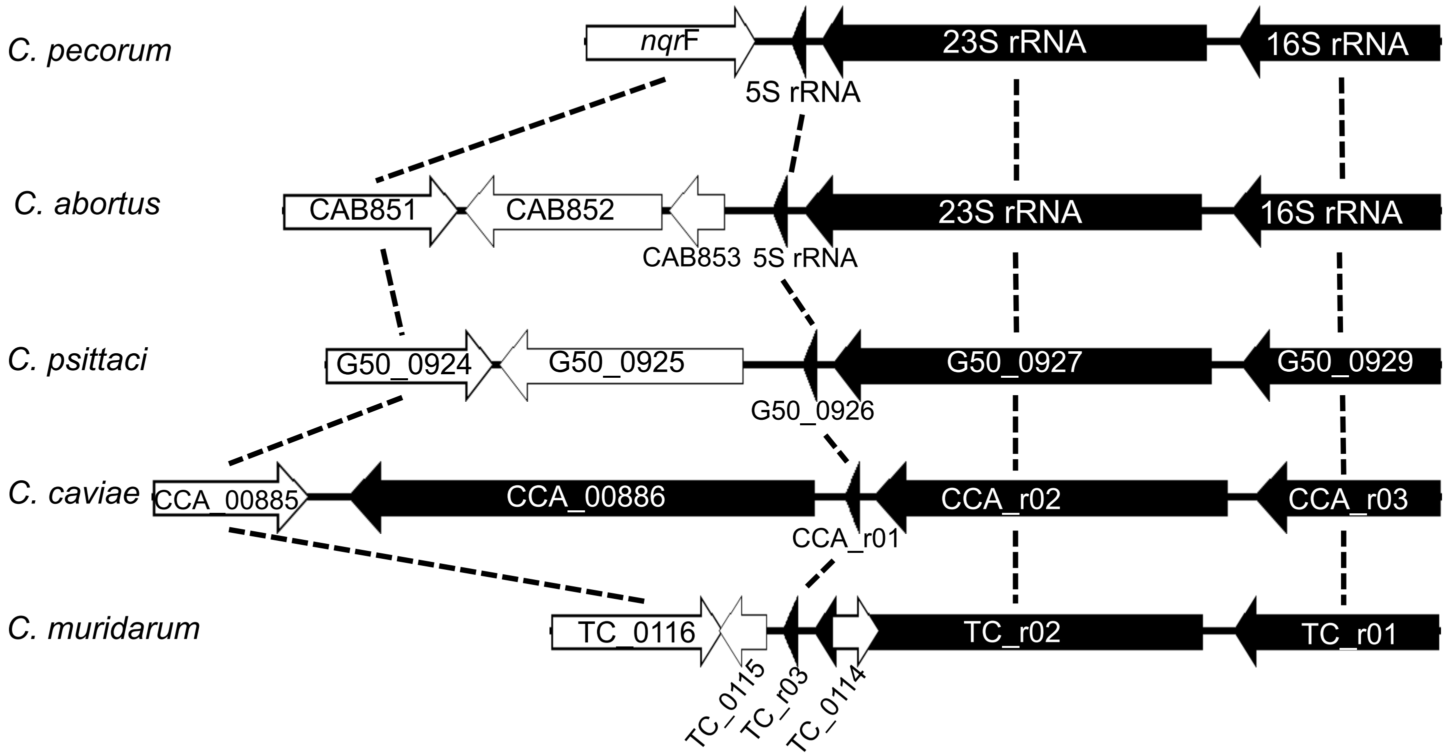

B

```
PV3056/3   AAAATAAGGC TATTTTCTAG -CTTTTTTTT ATAGACACTC TTTACCTCC- -------AAA GCACTAAAGC ACTAAAGCAC TAAAGCACTA AAGCACTAAA
W73        AAAATAGCCT TATTTTCTAG -CTTTTTTTT ATAGACACTC TTTACCTCC- -------AAA GCACTAAAGC ACTAAAGCAC TAAAGCACTA AAGCACTAAA
P787       AAAATAGCCT TATTTTCTAG -CTTTTTTTT ATAGACACTC TTTACCTCCA AAGCACTAAA GCACTAAAGC ACTAAAGCAC TAAAGCACTA AAGCACTAAA
E58        AAAATAGCCT TATTTTCTAG TTTTTTTTTT ATAGACACTC TTTACCTCC- -------AAA GCACTAAAGC ACTAAAGCAC TAAAGCACTA AAGCACTAAA

PV3056/3   GCACTAAAGC ACTAAAGCAC TAAAGCACTA AAGCACTAAA GCACTAAAGC ACTAAAGCAT AAAATTTAGT TGAGTCGCAG AAAAATATGA AAC-AAAAAA
W73        GCACTAAAGC ACTAAAGCAC TAAAGCACTA AAGCACTAAA GCACTAAAGC ACTAAAGCAT AAAATTTAGT TGAGTCGCAG AAAAATATGA AAC-AAAAAA
P787       GCACTAAAGC ACTAAAGCAC TAAAGCACTA AAGCACTAAA GCACTAAAGC ACTAAAGCAT AAAATTTAGT TGAGTCGCAG AAAAATATGA AAC-AAAAAA
E58        GCACTAAAGC ACTAAAGCAC TAAAGCACTA AAGCACTAAA GCACTAAAGC ACTAAAGCAT AAAATTTAGT TGAGTCGCAG AAAAATATGA AACAAAAAAA

PV3056/3   AAGAGAACAA AATCGCTTTT GTCCTCTTTT CTTAAACTTA GTCTTATGAC CAAGGAATCT TTTATATAAA A-
W73        AAGAGAACAA AATCGCTTTT GTCCTCTTTT CTTAAACTTA GTCTTATGAC CAAGGAATCT TTTATATAAA A-
P787       AAGAGAACAA AATCGCTTTT GTCCTCTTTT CTTAAACTTA GTCTTATGAC CAAGGAATCT TTTATATAAA A-
E58        AAGAGAACAA AATCGCTTTT GTCCTCTTTT CTTAAACTTA GTCTTATGAC CAAGGAATCT TTTATATAAA AA
```
